# Shared genetic architecture between Crohn’s disease and IgA nephropathy: implications for a gut–immune–kidney axis

**DOI:** 10.3389/fimmu.2026.1905767

**Published:** 2026-08-04

**Authors:** Tao Su, Mengjun Liang, Xiaohang Gao, Rui Lin, Hongzhen Wu, Luying Wu, Min Zhang, Tao Liu, Xiang Peng, Junzhang Zhao, Jun Deng, Jiayin Yao, Min Zhi

**Affiliations:** 1 Department of Gastroenterology, The Sixth Affiliated Hospital of Sun Yat-sen University, Guangzhou, Guangdong, China; 2 Guangdong Provincial Clinical Research Center for Digestive Diseases, Guangzhou, Guangdong, China; 3 Department of General Internal Medicine, The Sixth Affiliated Hospital of Sun Yat-sen University, Guangzhou, Guangdong, China; 4 Department of Radiation Oncology, Sun Yat-sen University Cancer Center, Guangzhou, Guangdong, China

**Keywords:** Crohn’s disease, genetic correlation, gut–immune–kidney axis, IgA nephropathy, Mendelian randomization

## Abstract

**Background:**

Inflammatory bowel disease (IBD) and IgA nephropathy (IgAN) share features of mucosal immune dysregulation, but the genetic basis of their relationship and the contribution of specific IBD subtypes remain unclear.

**Methods:**

Using public genome-wide association study (GWAS) summary datasets, we performed bidirectional Mendelian randomization (MR) analyses in European and East Asian populations, together with generalized summary-data-based Mendelian randomization (GSMR), Causal Analysis Using Summary Effect Estimates (CAUSE), genetic correlation, enrichment, cross-trait locus analysis, colocalization, and single-cell RNA sequencing. Phenotypic support was assessed in a clinical cohort of 9,371 patients with IBD.

**Results:**

Crohn’s disease (CD), but not ulcerative colitis (UC), showed a consistent directional genetic association with IgAN across populations, and GSMR provided complementary support for this association (P = 4.02 × 10^-5^). CAUSE did not clearly favor the causal model over the sharing model. Standard linkage disequilibrium score regression (LDSC) identified a positive genetic correlation between CD and IgAN (rg = 0.392, SE = 0.093, P = 2.53 × 10^-5^), with constrained-intercept LDSC and genetic covariance analyzer (GNOVA) providing supportive evidence. Functional analyses showed enrichment in whole blood and spleen, but not kidney tissue. Integrative analyses prioritized *ERAP2* and *CARD9* as leading candidate genes, with the strongest colocalization support observed for *ERAP2* in sigmoid-colon expression quantitative trait locus (eQTL) data (posterior probability of colocalization [PPH4] = 90.4%) and for *CARD9* at the GWAS level (PPH4 = 98.4%). In the clinical cohort, biopsy-confirmed IgAN was more common in CD than in UC (Firth-adjusted OR = 3.40, 95% CI 1.28–11.28; P = 0.0125).

**Conclusions:**

These findings support shared genetic architecture between CD and IgAN and suggest a possible directional genetic association between CD liability and IgAN risk. *ERAP2* and *CARD9* were prioritized as candidate genes within a proposed gut–immune–kidney framework.

## 1 Introduction

Inflammatory bowel disease (IBD) is a group of immune-mediated disorders characterized by chronic, relapsing inflammation of the gastrointestinal tract (1). Its two main subtypes are Crohn’s disease (CD) and ulcerative colitis (UC). Although IBD primarily affects the gut, it is increasingly recognized as a systemic disease with extraintestinal involvement (2). Growing attention has focused on the extraintestinal manifestations of IBD, driven by emerging insights into gut barrier dysfunction, altered antigen exposure, and immune dysregulation (3). IgA nephropathy (IgAN) is one of the most common primary glomerular diseases worldwide. It is associated with dysregulation of the gut immune system, aberrant IgA responses, and systemic immune activation (4, 5). These observations suggest that the gut may serve not only as a site of injury in IBD, but also as a driver of kidney injury through immune-mediated pathways (6, 7). Together, these findings support shared biological mechanisms between the two diseases and provide a rationale for investigating their link through the gut–kidney axis. Notably, targeted-release budesonide, which targets Peyer’s patches in the distal ileum, has been approved by the U.S. Food and Drug Administration for the treatment of IgAN (5).

Previous epidemiologic studies suggest a genuine link between IBD and IgAN (8). Cohort studies and real-world analyses have shown that patients with IBD can develop kidney involvement, including IgAN (9). Nakayama and colleagues further reported that IBD was associated with an approximately twofold higher risk of IgAN (7). Together, these findings support a shared disease process linking intestinal inflammation to kidney immune injury. Genetic evidence has also emerged, with early genome-wide association studies (GWAS) identifying overlap between IBD and IgAN at several immune-related loci and pathways (10, 11). Subsequent Mendelian randomization (MR) studies likewise suggested that IBD may increase the risk of IgAN (12, 13). However, important limitations remain. Observational studies are susceptible to residual confounding, detection bias, and variation in disease severity or treatment, while early genetic studies were largely restricted to shared loci or pathways and prior MR analyses used relatively limited analytic frameworks. Thus, it is still unclear whether IBD and IgAN share a common genetic basis, which genes are involved, and how this relationship is biologically mediated.

Building on this background, we systematically investigated the genetic relationship between IBD and IgAN. We examined IBD as a whole and its major subtypes separately, using a multimodal framework that included MR, genome-wide genetic correlation, functional enrichment, local genetic architecture, colocalization, summary-data-based MR (SMR), and single-cell transcriptomics. We further sought to identify the key genes underlying this link and the immune cell types in which they are most active, and to assess phenotypic support for the main findings in a clinical cohort. Together, these analyses were intended to define the IBD–IgAN relationship from genetic, functional, and clinical perspectives.

## 2 Materials and methods

### 2.1 Data sources and study overview

We integrated publicly available GWAS summary statistics, expression quantitative trait locus (eQTL) resources, and a published single-cell RNA sequencing dataset. All genetic analyses were based on summary-level data, and no individual-level genotypes were accessed. Detailed information on all datasets is provided in **Supplementary Table 1**, and additional analytic details are provided in the **Supplementary Methods** (see **Supplementary Material**).

For IBD, CD, and UC, we used large GWAS summary datasets from European and East Asian populations, where available (14, 15). For IgAN, we used the primary GWAS datasets from the Kiryluk laboratory (11). IgAN cases in these datasets were biopsy-confirmed, improving diagnostic specificity for downstream analyses. Bidirectional MR analyses included both European and East Asian datasets where available, whereas most downstream analyses were based on European datasets.

To investigate expression-related mechanisms, we used whole-blood eQTL data from eQTLGen and tissue-specific eQTL data from GTEx v8, including whole blood, spleen, terminal ileum, sigmoid colon, transverse colon, lung, and liver (16, 17). These datasets were used for SMR and, where available, colocalization analyses. To define the cellular context of prioritized genes, we included the nine-donor peripheral blood mononuclear cell (PBMC) subset of the published GSE134809 single-cell RNA-sequencing dataset from patients with CD (18). Potential sample overlap between GWAS datasets was evaluated in the genome-wide genetic correlation analyses.

### 2.2 Bidirectional Mendelian randomization, GSMR, and CAUSE

To assess potential causal relationships between IBD, its major subtypes, and IgAN, we performed bidirectional two-sample MR analyses, with generalized summary-data-based MR (GSMR) and causal analysis using summary effect estimates (CAUSE) as complementary approaches (19). Forward analyses evaluated IBD, CD, and UC as exposures with IgAN as the outcome, whereas reverse analyses used IgAN as the exposure and each IBD subtype as the outcome. Both directions were examined in European and East Asian populations where data were available.

Instrumental variables were selected at the genome-wide significance threshold (P < 5 × 10^-8^) and pruned for linkage disequilibrium to retain independent variants. The inverse-variance weighted (IVW) method served as the primary estimator. MR-Egger, weighted median, weighted mode and simple mode analyses were used as sensitivity analyses, together with the MR-Egger intercept test, Cochran’s Q test, and MR-PRESSO (20–22). Detailed procedures for instrument selection and harmonization, Steiger directionality testing, sensitivity analyses, and multiple-testing correction are provided in the **Supplementary Methods**.

Given the consistent CD–IgAN signal observed in bidirectional MR and genome-wide genetic correlation analyses, GSMR and CAUSE were applied specifically to this disease pair in the European population. GSMR used the HEIDI-outlier approach to reduce bias from horizontal pleiotropy (23). The number of retained instruments was considered when interpreting differences in precision and statistical power between the forward and reverse estimates. CAUSE compared sharing and causal models to distinguish directional effects from associations driven by shared genetic architecture (24). Because CD and IgAN likely share a broad immune-genetic background, these analyses were interpreted as robustness checks rather than primary evidence for causality.

### 2.3 Genome-wide genetic correlation analyses: LDSC and GNOVA

To assess the genome-wide shared genetic basis between IBD, CD, UC, and IgAN, we performed genetic correlation analyses using linkage disequilibrium score regression (LDSC) and genetic covariance analyzer (GNOVA) based on European GWAS summary statistics.

Using European reference LD scores, LDSC was applied to estimate single nucleotide polymorphism (SNP) heritability and pairwise genetic correlation (rg) (25). We also used the cross-trait intercept to evaluate potential sample overlap between GWAS datasets (26).

We then applied GNOVA to estimate genetic covariance and genetic correlation between trait pairs, and results were reported before and after correction to assess robustness (27). Findings from LDSC and GNOVA were interpreted together to evaluate the direction and strength of the shared genetic architecture between IBD-related traits and IgAN.

### 2.4 Functional enrichment and tissue/pathway analyses

To characterize the shared genetic basis of CD and IgAN at the functional level, all annotation and enrichment analyses were conducted using European GWAS summary statistics. We applied stratified linkage disequilibrium score regression (S-LDSC), linkage disequilibrium score regression for specifically expressed genes (LDSC-SEG), and multi-marker analysis of genomic annotation (MAGMA) to evaluate enrichment across functional annotations, tissues, and biological pathways.

S-LDSC was performed using the baselineLD v2.2 annotation set to assess heritability enrichment across functional categories (28). Tissue-specific enrichment was assessed using both LDSC-SEG and MAGMA based on GTEx tissue expression data (29, 30). Signals that remained significant after multiple-testing correction were prioritized, whereas consistent but non-significant patterns across methods were treated as supportive evidence.

MAGMA was also used for competitive gene-set analysis in CD and IgAN using 17,009 gene sets from the Molecular Signatures Database (MSigDB; v2023.1.Hs). Multiple testing was controlled separately within each trait using the Benjamini–Hochberg false discovery rate (BH-FDR). Pathway findings were interpreted in the context of the tissue-enrichment results.

### 2.5 Cross-trait locus discovery: PLACO and MTAG

To identify potential shared loci between CD and IgAN, we performed cross-trait analyses using pleiotropic analysis under composite null hypothesis (PLACO) and multi-trait analysis of genome-wide association studies (MTAG) based on European GWAS summary statistics (31, 32). MTAG was used to improve locus discovery by leveraging the genetic correlation between the two traits, whereas PLACO was used to test whether the same variant was associated with both traits. Together, these methods provided complementary trait-specific and cross-trait association evidence.

MTAG was performed using standardized GWAS summary statistics, with potential sample overlap accommodated by estimating the error covariance matrix. Trait-specific mean χ² values, genetic and error covariance matrices, GWAS-equivalent sample sizes, and maximum FDR values were obtained as diagnostic measures. PLACO was applied after decorrelation of the trait-specific Z statistics using their estimated cross-trait correlation (31, 32). Potential additional shared loci were defined from PLACO-significant variants, followed by linkage disequilibrium (LD)-based pruning using the European 1000 Genomes reference panel. MTAG results were used to characterize trait-specific association evidence but were not used alone to define shared loci. Variants already supported as genome-wide significant in both original GWAS were treated as known shared loci, and the remaining independent PLACO-significant variants were retained as potential additional shared loci (33).

### 2.6 Local genetic architecture analysis: LAVA

To assess the local shared genetic architecture between CD and IgAN, we performed local genetic correlation analysis using local analysis of [co]variant association (LAVA) (34). Analyses were based on European GWAS summary statistics and the European 1000 Genomes reference panel, with local regions defined using the LAVA semi-independent LD block framework derived from Berisa and Pickrell (35).

Before running LAVA, we used LDSC to estimate the global genetic correlation between the two traits and to account for potential sample overlap (26).

LAVA was then applied in a two-step framework, with bivariate local genetic correlation analysis performed only in LD blocks that passed the prespecified univariate screening step. Statistical significance for bivariate results was determined using a Bonferroni-corrected threshold. These results were used to map the nonuniform distribution of local genetic correlation across the genome and to nominate candidate regions for subsequent colocalization analysis.

### 2.7 Colocalization and summary-data-based Mendelian randomization

To assess whether CD and IgAN share causal variants and whether shared signals might operate through gene expression regulation, we performed SMR and colocalization analyses using European GWAS summary statistics together with whole-blood eQTL data from eQTLGen and tissue-specific eQTL data from GTEx v8, where appropriate.

SMR was used to test whether disease-associated signals might act through differences in gene expression, with the HEIDI test used to distinguish a shared causal variant from linkage disequilibrium-driven associations (36).

Candidate regions for colocalization were defined based on loci showing significant local genetic correlation in LAVA and signals highlighted by SMR. We performed both GWAS–GWAS and GWAS–eQTL colocalization analyses using the coloc five-hypothesis framework, with the posterior probability of a shared causal variant (PPH4) as the primary criterion (37). SMR and colocalization results were interpreted together to prioritize candidate genes for downstream functional analyses.

### 2.8 Single-cell RNA sequencing analysis

To define the cell-type distribution of candidate genes and their immune-cell context, we analyzed the nine PBMC samples from GSE134809; ileal lamina propria samples from the original study were not included (18). Deposited 10x Genomics count matrices were used for downstream quality control, batch correction, clustering, and annotation, as detailed in the **Supplementary Methods** (38, 39).

The single-cell analysis was used to characterize the expression patterns of candidate genes across annotated immune cell types and to provide cell-type-level support for the genetic findings. The main-text results focus on the composition of annotated immune cell populations after quality control and the expression patterns of candidate genes across key cell types.

### 2.9 Retrospective clinical cohort

To assess phenotypic support for the genetic findings at the clinical level, we assembled a single-center retrospective cohort using the inpatient medical record system of the Sixth Affiliated Hospital of Sun Yat-sen University. All hospitalized patients with a confirmed diagnosis of CD or UC between October 2012 and October 2025 were considered for inclusion. Demographic information, diagnostic records, and kidney-related clinical data were extracted for analysis.

Patients were included if they had a definite diagnosis of CD or UC with sufficient documentation to support IBD subtype classification (40). Individuals with ambiguous or provisional diagnoses were excluded, as were those in whom IgAN predated the IBD diagnosis. The primary outcome was biopsy-confirmed IgAN, and the secondary outcome was any biopsy-confirmed renal parenchymal disease (41). Nephrolithiasis, renal dysfunction without pathological confirmation, and non-parenchymal or urological conditions were not included in the secondary outcome. Outcome frequencies were compared between CD and UC using contingency tables. Crude comparisons were assessed using Fisher’s exact test, and Firth bias-reduced logistic regression was used to estimate adjusted odds ratios for CD relative to UC after adjustment for age and sex. Corresponding 95% confidence intervals were derived from the penalized profile likelihood, and adjusted P values were obtained using penalized likelihood ratio tests. Operational definitions of the exposures, renal outcomes, and covariates are provided in **Supplementary Table 5**.

The study was approved by the Ethics Committee of The Sixth Affiliated Hospital, Sun Yat-sen University [approval number: 2026ZSLYEC-364], which waived the requirement for written informed consent because of the retrospective design.

### 2.10 Software and statistical environment

Unless otherwise specified, statistical analyses were performed in R. Single-cell RNA sequencing analyses were conducted using Seurat and scDblFinder. Other analyses were performed using established tools for Mendelian randomization, GSMR, CAUSE, LDSC-related analyses, colocalization, local genetic correlation, tissue and pathway enrichment, PLACO, and SMR.

## 3 Results

### 3.1 Directional genetic association of Crohn’s disease liability with IgA nephropathy risk

**Figure 1:**
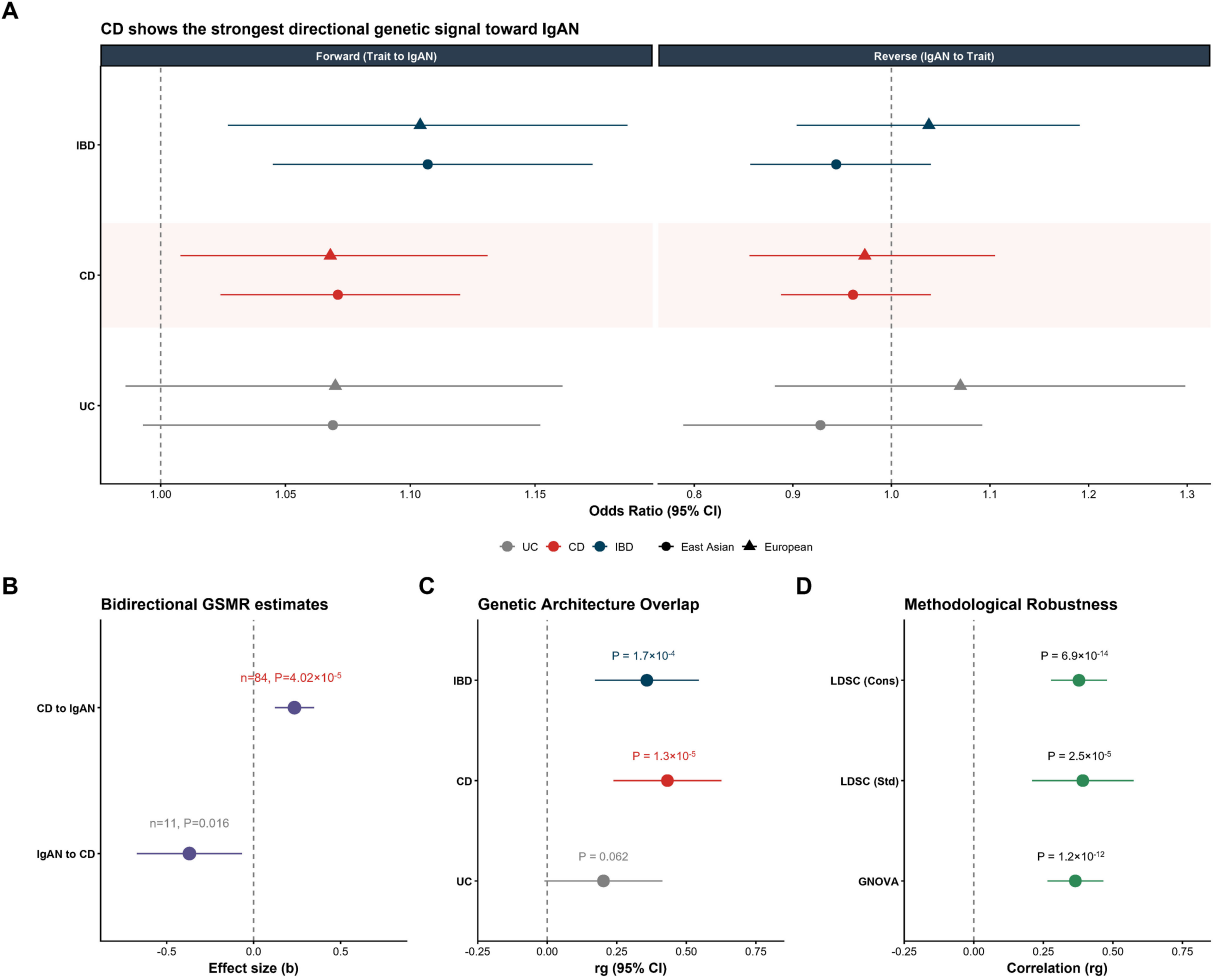
Crohn’s disease exhibits the strongest directional genetic and genome-wide genetic-correlation signal with IgA nephropathy. **(A)** Bidirectional two-sample MR analyses of IBD, CD, and UC in relation to IgAN in European and East Asian populations. Points denote ORs and horizontal lines denote 95% CIs. Colors indicate IBD subtype, and point shapes indicate ancestry. The vertical dashed line indicates OR = 1. **(B)** Bidirectional GSMR estimates for the CD–IgAN pair, shown as effect-size estimates **(b)** with 95% CIs, number of instrumental variants, and P values for the forward and reverse directions. The vertical dashed line indicates b = 0. **(C)** Genome-wide genetic correlations between IgAN and the three IBD phenotypes. **(D)** Cross-method comparison of the CD–IgAN genetic correlation estimated by GNOVA and standard and constrained LDSC. In **(C, D)**, points denote correlation estimates, horizontal lines denote 95% CIs, and the vertical dashed line indicates rg = 0. CD, Crohn’s disease; CI, confidence interval; GNOVA, genetic covariance analyzer; GSMR, generalized summary-data-based Mendelian randomization; IBD, inflammatory bowel disease; IgAN, IgA nephropathy; LDSC, linkage disequilibrium score regression; MR, Mendelian randomization; OR, odds ratio; rg, genetic correlation; UC, ulcerative colitis.

Bidirectional two-sample MR showed that genetic liability to CD was associated with an increased risk of IgAN, with a consistent direction of effect in both European and East Asian populations (**Figure 1A**). In the primary IVW analysis, the odds ratio (OR) for CD→IgAN was 1.068 (95% CI 1.008–1.131, P = 0.0248, BH-FDR = 0.0372) in Europeans and 1.071 (95% CI 1.024–1.120, P = 0.0027, BH-FDR = 0.0082) in East Asians. Overall IBD also showed positive associations that remained significant after BH-FDR correction in both populations, whereas UC was not statistically significant in either ancestry, indicating that CD was the subtype most clearly associated with IgAN. Full two-sample MR results, including instrument counts at each stage of variant selection, F statistics, variance explained, Steiger directionality results, and complete sensitivity-analysis results, are provided in **Supplementary Table 2**.

Reverse MR analyses did not identify a consistent genetic effect of IgAN on overall IBD or any of its subtypes across populations, and the IVW estimates were not significant in either the European or East Asian dataset. Bidirectional GSMR yielded a positive CD→IgAN estimate (b = 0.235, n = 84, P = 4.02 × 10^-5^) and a negative IgAN→CD estimate (b = −0.369, n = 11, P = 0.0165). Because the reverse GSMR analysis retained substantially fewer instruments, its estimate was less precise, and the difference between the two directions was interpreted cautiously. Taken together with the cross-ancestry two-sample MR results, the overall evidence was more consistent with a CD→IgAN association, whereas the reverse direction remained uncertain (**Figure 1B**).

Because MR estimates may still be influenced by shared genetic architecture, we further performed CAUSE as a sensitivity analysis. CAUSE did not provide stronger support for a causal model than for a sharing model, indicating that shared genetic architecture may contribute to the observed CD–IgAN association(**Supplementary Figure 1**).

### 3.2 Genome-wide polygenic sharing between Crohn’s disease and IgA nephropathy

To determine whether the CD–IgAN association reflects a broader shared genetic basis, we next evaluated genome-wide genetic correlation between the two diseases. LDSC showed a significant positive genetic correlation between CD and IgAN (rg = 0.392, SE = 0.093, P = 2.53 × 10^-5^), which remained robust after constraining the intercept (rg = 0.378, SE = 0.051, P = 6.94 × 10^-^¹^4^). GNOVA showed the same pattern, with estimates of 0.365 before correction (SE = 0.051, P = 1.21 × 10^-^¹²) and 0.274 after correction (SE = 0.049, P = 2.89 × 10^-8^). The effect sizes were highly concordant across all three analyses (**Figure 1D**), indicating that CD and IgAN share a broad polygenic architecture that coexists with the directional association observed in the MR analyses (**Figure 1C**). By contrast, the genetic correlation between UC and IgAN did not reach statistical significance (P = 0.062), further suggesting that shared polygenic susceptibility within IBD is more pronounced for CD than for UC.

### 3.3 Immune tissue and pathway enrichment of shared genetic liability

**Figure 2:**
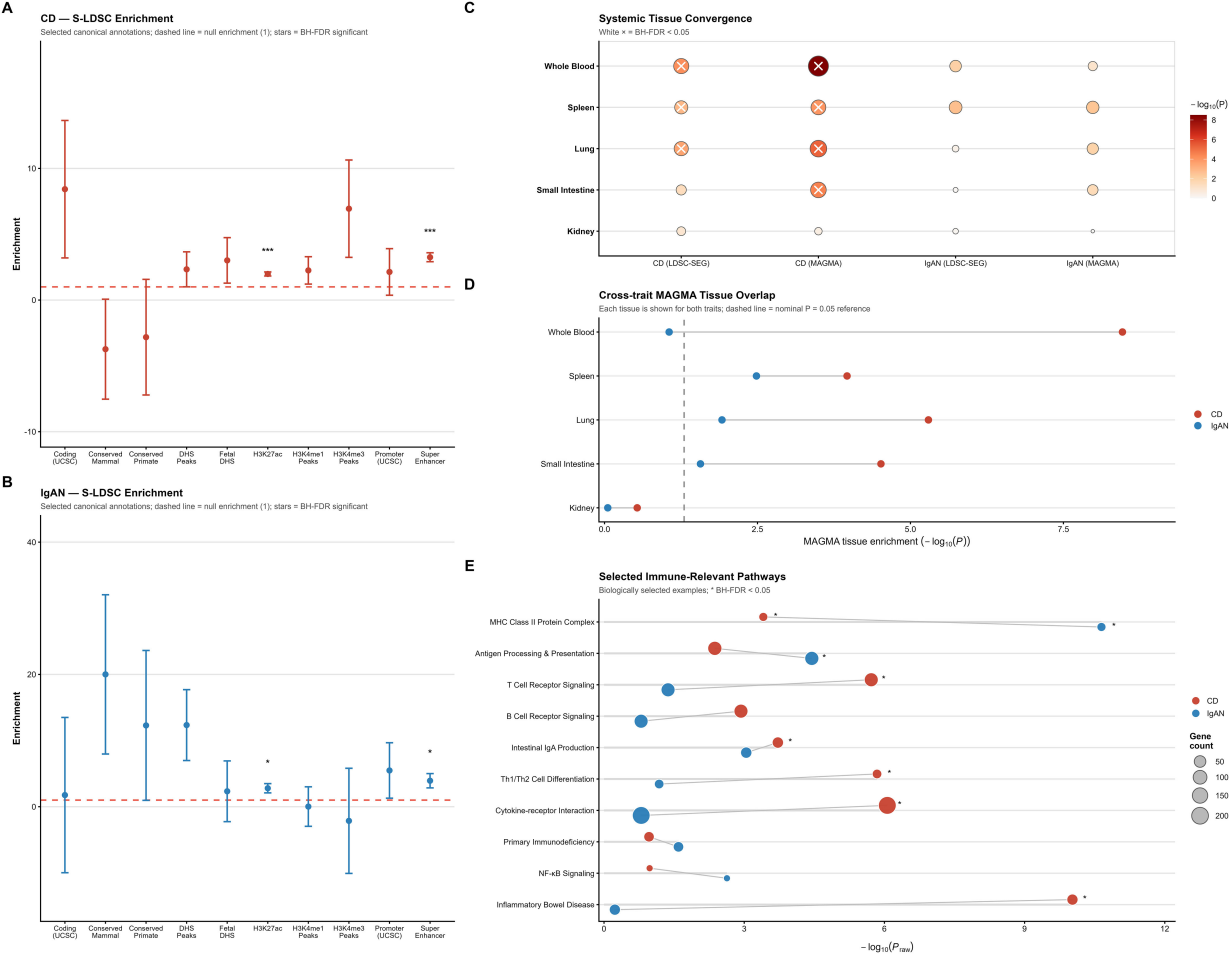
Functional enrichment, tissue convergence, and shared immune pathways linking Crohn’s disease and IgA nephropathy. **(A, B)** S-LDSC enrichment across selected canonical genomic annotations in CD **(A)** and IgAN **(B)**. Points denote enrichment estimates and vertical lines denote 95% CIs. The horizontal dashed line indicates enrichment = 1, and asterisks indicate annotations with BH-FDR < 0.05. **(C)** Tissue-level convergence between CD and IgAN across selected systemic and disease-relevant tissues, integrating LDSC-SEG and MAGMA results. Circle color intensity denotes statistical significance [−log10(P)], and white crosses indicate tissue–method pairs passing multiple-testing correction. **(D)** Cross-trait comparison of MAGMA tissue-enrichment signals for CD and IgAN across selected tissues. The vertical dashed line indicates the nominal significance threshold. **(E)** Selected immune-relevant pathways in CD and IgAN. Point position denotes −log10(P), point size denotes the number of mapped genes, and asterisks indicate BH-FDR < 0.05; unmarked pathways were selected on biological grounds. CD, Crohn’s disease; CI, confidence interval; DHS, DNase I hypersensitive site; IgAN, IgA nephropathy; LDSC-SEG, linkage disequilibrium score regression for specifically expressed genes; MAGMA, Multi-marker Analysis of GenoMic Annotation; NF-κB, nuclear factor kappa B; S-LDSC, stratified linkage disequilibrium score regression.

To define the functional context of the shared genetic liability between CD and IgAN, we partitioned heritability across genomic annotations. S-LDSC showed enrichment of genetic signals for both diseases in active regulatory elements, particularly enhancers and open chromatin regions, implicating noncoding immune-regulatory regions (**Figures 2A, B**).

At the tissue level, LDSC-SEG and MAGMA showed the same overall pattern, pointing to systemic immune tissues rather than kidney-localized tissues (**Figures 2C, D**). For CD, LDSC-SEG identified significant enrichment after multiple-testing correction in whole blood (P = 6.62 × 10^-5^) and lung (P = 2.90 × 10^-4^), with nominal enrichment also observed in spleen (P = 1.30 × 10^-^³); MAGMA gave a consistent result, with the strongest signal in whole blood. For IgAN, spleen and whole blood were nominally significant in LDSC-SEG (P = 2.05 × 10^-^³ and P = 8.89 × 10^-^³, respectively), and spleen showed concordant enrichment in MAGMA (P = 3.29 × 10^-^³), although the overall signals were weaker and did not survive stringent multiple-testing correction. In contrast, kidney cortex showed no clear enrichment in either disease by either method, suggesting that the shared genetic liability is not primarily driven by kidney-specific regulatory programs.

At the pathway level, MAGMA gene-set analysis identified 350 and 15 gene sets meeting BH-FDR < 0.05 in CD and IgAN, respectively. **Figure 2E** presents a biologically selected subset of immune- and mucosal-relevant pathways, including antigen processing and presentation, MHC class II protein complex, T cell and B cell receptor signaling, intestinal IgA production, cytokine–receptor interaction, and NF-κB signaling. Asterisks denote trait-specific BH-FDR significance, whereas unmarked pathways were included for biological relevance but did not meet the corrected significance threshold. Complete results for all 17,009 tested gene sets, including database sources, raw P values, BH-FDR values, and Bonferroni-adjusted P values, are provided in **Supplementary Table 6**. Together, these findings place the shared CD–IgAN genetic architecture in systemic immune and mucosal immune contexts.

### 3.4 Local genetic architecture identifies shared susceptibility regions

**Figure 3:**
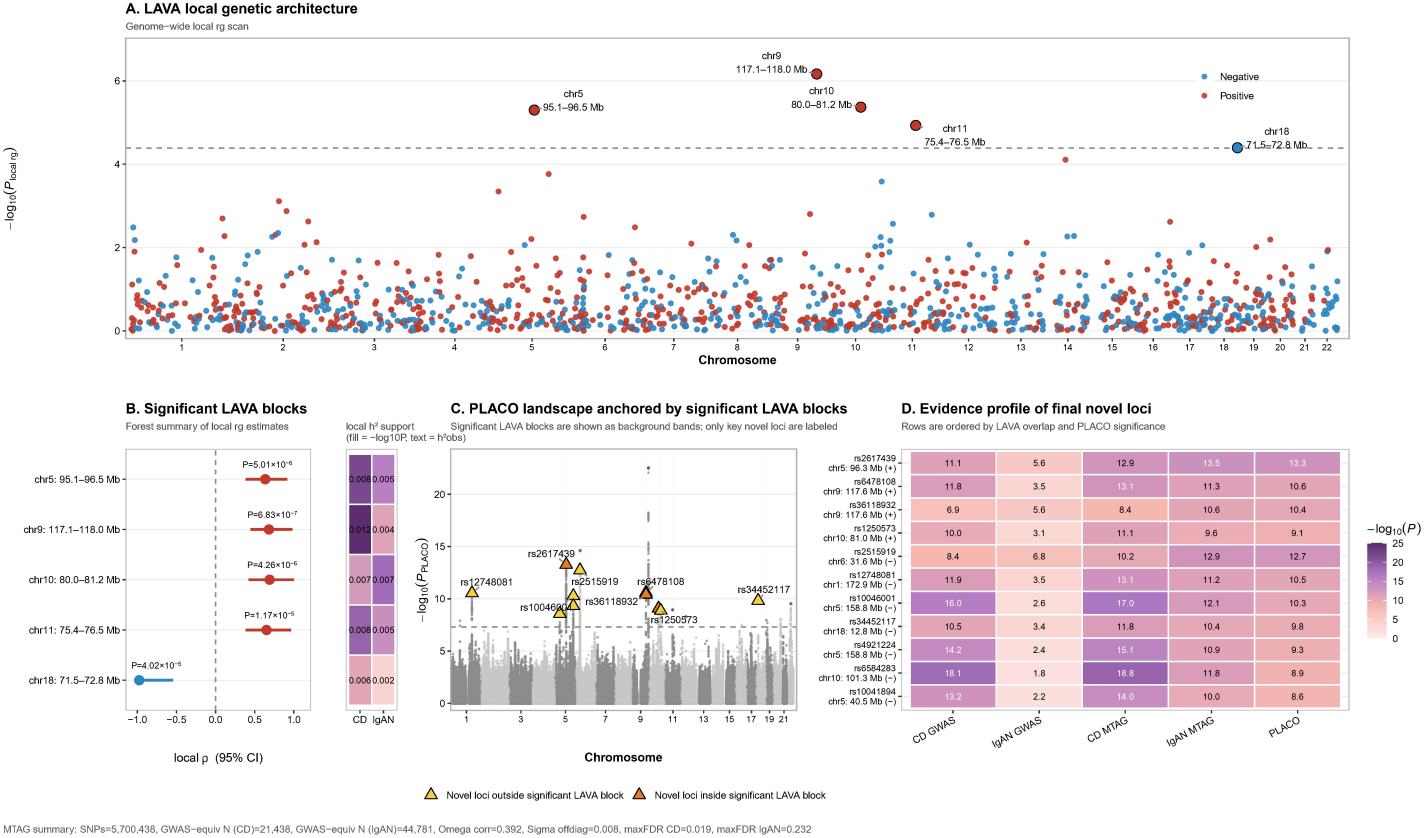
Local genetic architecture and additional shared-locus discovery. **(A)** Genome-wide scan of local genetic correlation across predefined genomic blocks using LAVA. Each point represents one block plotted by chromosomal position and −log10(P), with point color indicating the direction of local genetic correlation. The horizontal dashed line indicates the Bonferroni significance threshold, and significant blocks are labeled by chromosomal interval. **(B)** Forest summary of Bonferroni-significant LAVA blocks, showing local genetic-correlation estimates (ρ) with 95% CIs. The adjacent heatmap summarizes local SNP-based heritability support for CD and IgAN; fill denotes −log10(P), and text denotes observed local h² values. **(C)** Genome-wide PLACO landscape anchored by significant LAVA blocks. Background bands denote Bonferroni-significant LAVA regions, and highlighted triangles denote retained independent potential additional shared loci after significance filtering and LD-based pruning. Representative lead variants are labeled. **(D)** Integrated evidence profile of the retained potential additional shared loci across the original GWAS, MTAG, and PLACO analyses. Rows correspond to retained lead variants, and columns summarize significance profiles in CD GWAS, IgAN GWAS, CD MTAG, IgAN MTAG, and PLACO. Cell values and fill denote −log10(P). CD, Crohn’s disease; CI, confidence interval; GWAS, genome-wide association study; h², SNP-based heritability; IgAN, IgA nephropathy; LAVA, Local Analysis of [co]Variant Association; LD, linkage disequilibrium; MTAG, multi-trait analysis of GWAS; PLACO, pleiotropic analysis under composite null hypothesis; ρ, local genetic correlation.

To further resolve the local structure of shared genetic risk between CD and IgAN, we used LAVA to estimate local genetic correlation across the genome. The shared genetic architecture was not uniformly distributed, but instead concentrated in several discrete genomic regions. After Bonferroni correction, five significant local rg blocks were identified at chr5:95.1–96.5 Mb, chr9:117.1–118.0 Mb, chr10:80.0–81.2 Mb, chr11:75.4–76.5 Mb, and chr18:71.5–72.8 Mb. The chr5, chr9, chr10, and chr11 regions showed positive local genetic correlation, whereas chr18 showed a negative local genetic correlation, indicating both concordant shared-risk regions and regions with opposing local genetic correlation between CD and IgAN (**Figures 3A, B**).

PLACO and MTAG were then used to extend cross-trait locus discovery. PLACO identified putative shared signals both within and outside the LAVA-significant regions, with several loci on chr5, chr9, and chr10 overlapping Bonferroni-significant LAVA blocks. MTAG provided enhanced trait-specific association evidence at the PLACO-prioritized loci; its diagnostic statistics and covariance estimates are reported in **Supplementary Table 3** (**Figures 3C, D**). These regions and candidate loci were then carried forward into SMR and colocalization analyses to prioritize effector genes.

### 3.5 Integrative transcriptomic analyses prioritize *ERAP2* and *CARD9*

On the basis of candidate regions and genes jointly defined by LAVA-significant local genetic correlation and SMR results, we next performed integrative transcriptomic analyses to move from shared genomic regions to putative effector genes. SMR identified several candidate expression-regulated genes, among which *ERAP2* and *CARD9* showed the most coherent cross-disease evidence.

**Figure 4:**
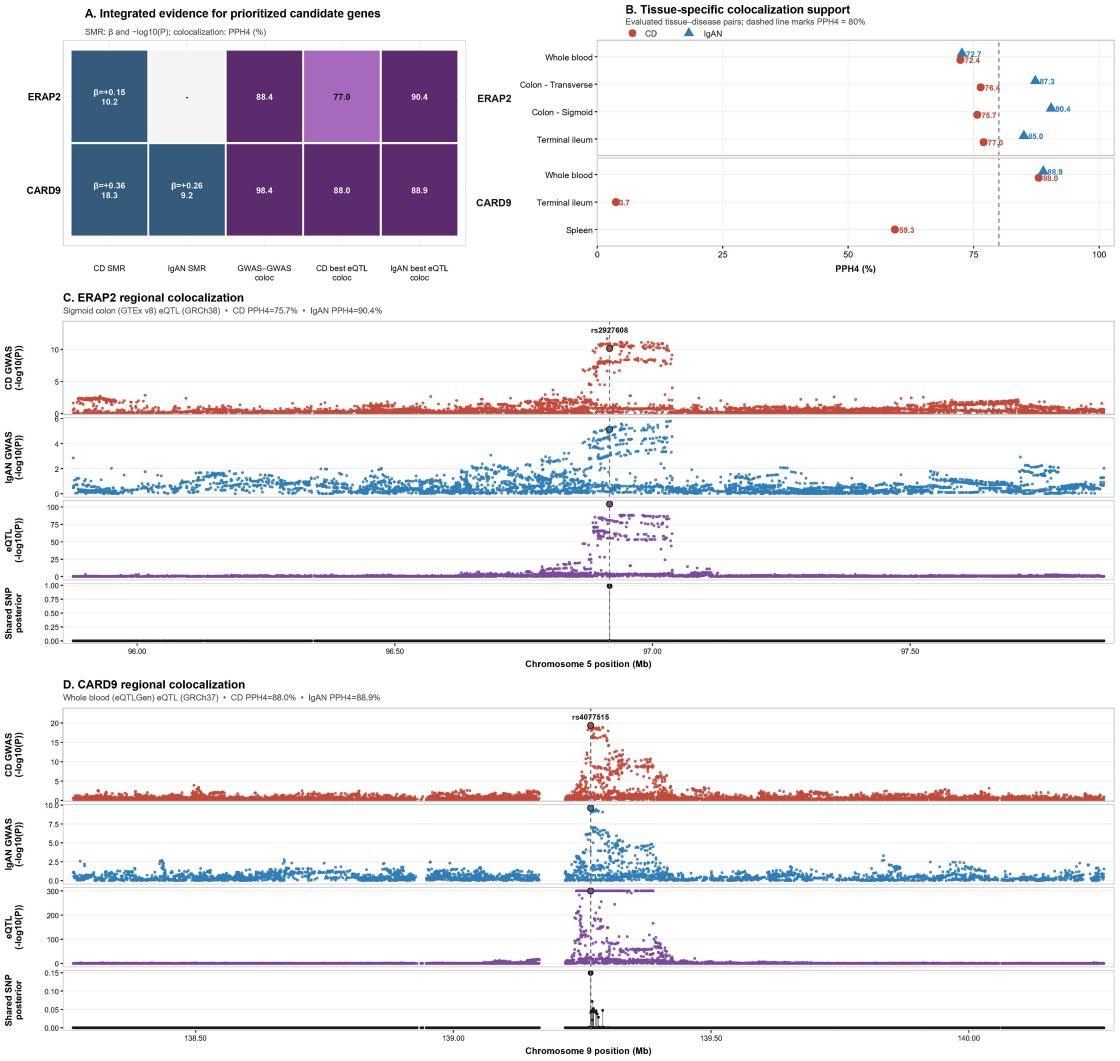
Integrative SMR and colocalization prioritize candidate genes at shared loci. **(A)** Integrated prioritization of candidate genes based on SMR and colocalization evidence. Cell values denote −log10 adjusted P values for SMR or PPH4 (%) for colocalization. **(B)** Tissue-resolved colocalization support for *ERAP2* and *CARD9* across the analyzed eQTL resources and tissues. Cell values denote PPH4 (%), and blank cells indicate tissue–disease pairs not analyzed. **(C, D)** Representative regional colocalization plots for *ERAP2* in sigmoid-colon eQTL data from GTEx v8 (GRCh38) **(C)** and *CARD9* in whole-blood eQTL data from eQTLGen (GRCh37) **(D)**. From top to bottom, the stacked tracks show association signals for CD, IgAN, the corresponding eQTL, and posterior support for shared causal-variant configurations across the locus. Annotated PPH4 values summarize colocalization support for CD and IgAN with the corresponding eQTL signal. CD, Crohn’s disease; eQTL, expression quantitative trait locus; GTEx, Genotype-Tissue Expression; IgAN, IgA nephropathy; PPH4, posterior probability for hypothesis 4; SMR, summary-data-based Mendelian randomization.

*ERAP2* mapped to a LAVA-supported shared region on chromosome 5. On the CD side, *ERAP2* showed significant SMR associations across multiple tissues, including whole blood, spleen, terminal ileum, and colon, with no clear evidence of heterogeneity by HEIDI. Although *ERAP2* did not reach the SMR significance threshold for IgAN, tissue-resolved GWAS–eQTL colocalization provided complementary support, with IgAN-side PPH4 values of 72.7%, 85.0%, 90.4%, and 87.3% in whole blood, terminal ileum, sigmoid colon, and transverse colon, respectively. GWAS–GWAS colocalization also supported a shared causal variant between CD and IgAN at this locus (PPH4 = 88.4%). Effect directions for *ERAP2* and alternative genes at the locus, including *CAST*, *ERAP1*, and *LNPEP*, together with the corresponding SMR and HEIDI results, are provided in **Supplementary Table 4**. Together, these findings prioritized *ERAP2* as a candidate gene at the 5q15 locus, supported by CD-side SMR and colocalization evidence. *CARD9* showed strong SMR signals in both CD and IgAN. Its GWAS–eQTL colocalization support was strongest in whole blood (CD PPH4 = 88.0%; IgAN PPH4 = 88.9%), with GWAS–GWAS colocalization reaching a PPH4 of 98.4% and additional moderate support in spleen (CD PPH4 = 59.3%). Integrating these layers of evidence, *ERAP2* and *CARD9* were prioritized as leading candidate genes (**Figures 4A–D**; **Supplementary Figure 2**; **Supplementary Table 4**).

**Figure 5:**
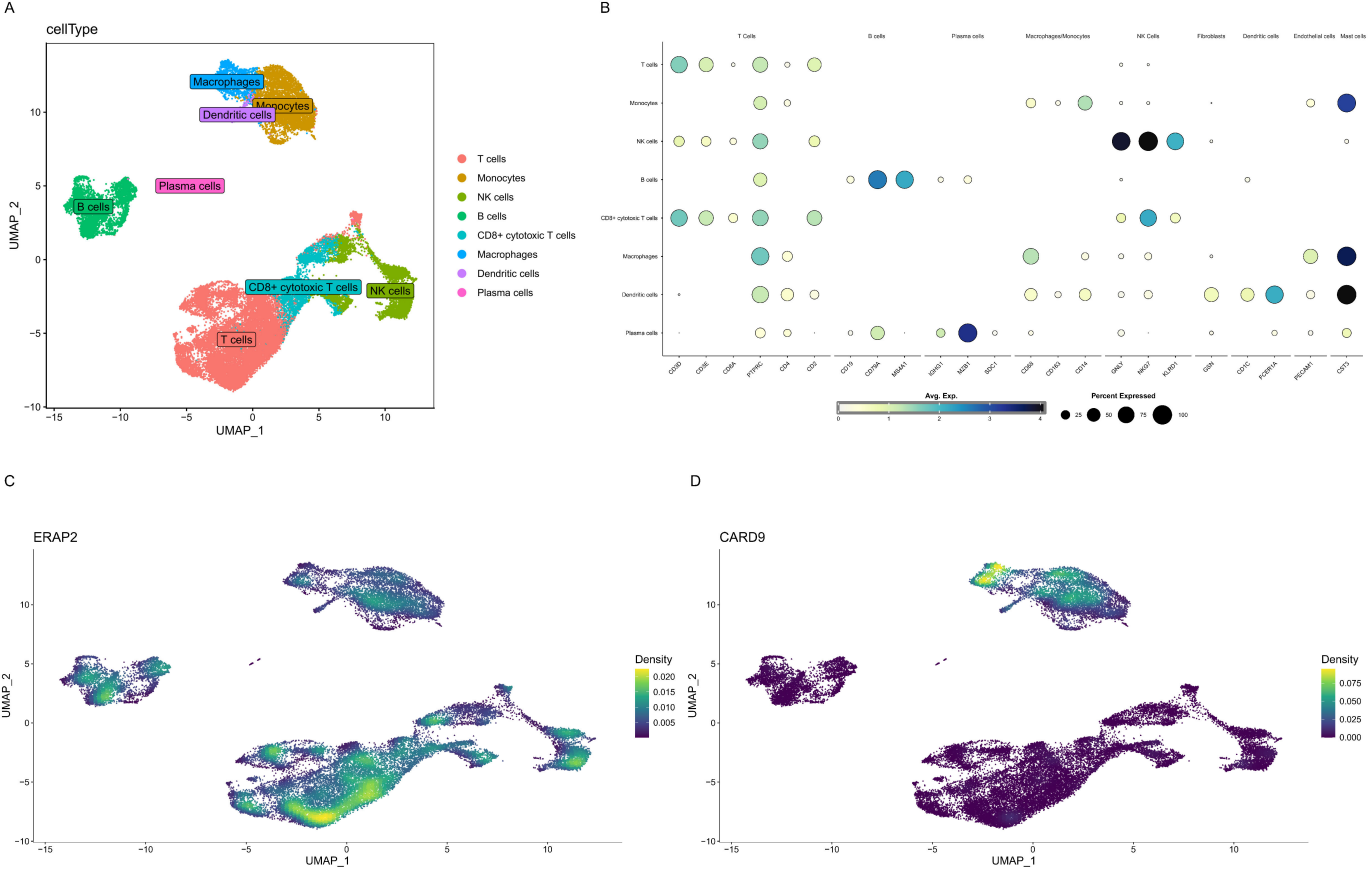
Single-cell immune atlas localizes *ERAP2* and *CARD9* to distinct immune compartments. **(A)** UMAP embedding of the curated immune-cell atlas derived from the PBMC subset of GSE134809, showing the retained immune-cell subsets in the final atlas. **(B)** Marker-based annotation panel validating the annotated immune-cell subsets. Dot color denotes average expression level, and dot size denotes the proportion of cells expressing the corresponding marker gene within each population. **(C)** Density-based feature visualization of *ERAP2* expression across the immune-cell atlas, showing broad distribution across immune compartments. **(D)** Density-based feature visualization of *CARD9* expression across the same atlas, showing enrichment in myeloid-associated compartments, particularly macrophage, monocyte, and dendritic cell clusters. CD, Crohn’s disease; NK, natural killer; PBMC, peripheral blood mononuclear cell; scRNA-seq, single-cell RNA sequencing; UMAP, uniform manifold approximation and projection.

Single-cell analysis provided additional cell-type context. In the PBMC subset of GSE134809, UMAP clustering identified major immune populations, including T cells, B cells, NK cells, monocytes, macrophage-like cells, dendritic cells, and plasma cells (**Figures 5A, B**). *ERAP2* was broadly expressed across multiple immune cell populations, whereas *CARD9* expression was concentrated in myeloid populations, including monocytes, macrophage-like cells, and dendritic cells, consistent with its role in NF-κB-associated myeloid inflammation (**Figures 5C, D**). The same annotated cells are shown using t-SNE as an alternative two-dimensional embedding in **Supplementary Figure 3**.

### 3.6 An integrative gut–immune–kidney axis framework

**Figure 6:**
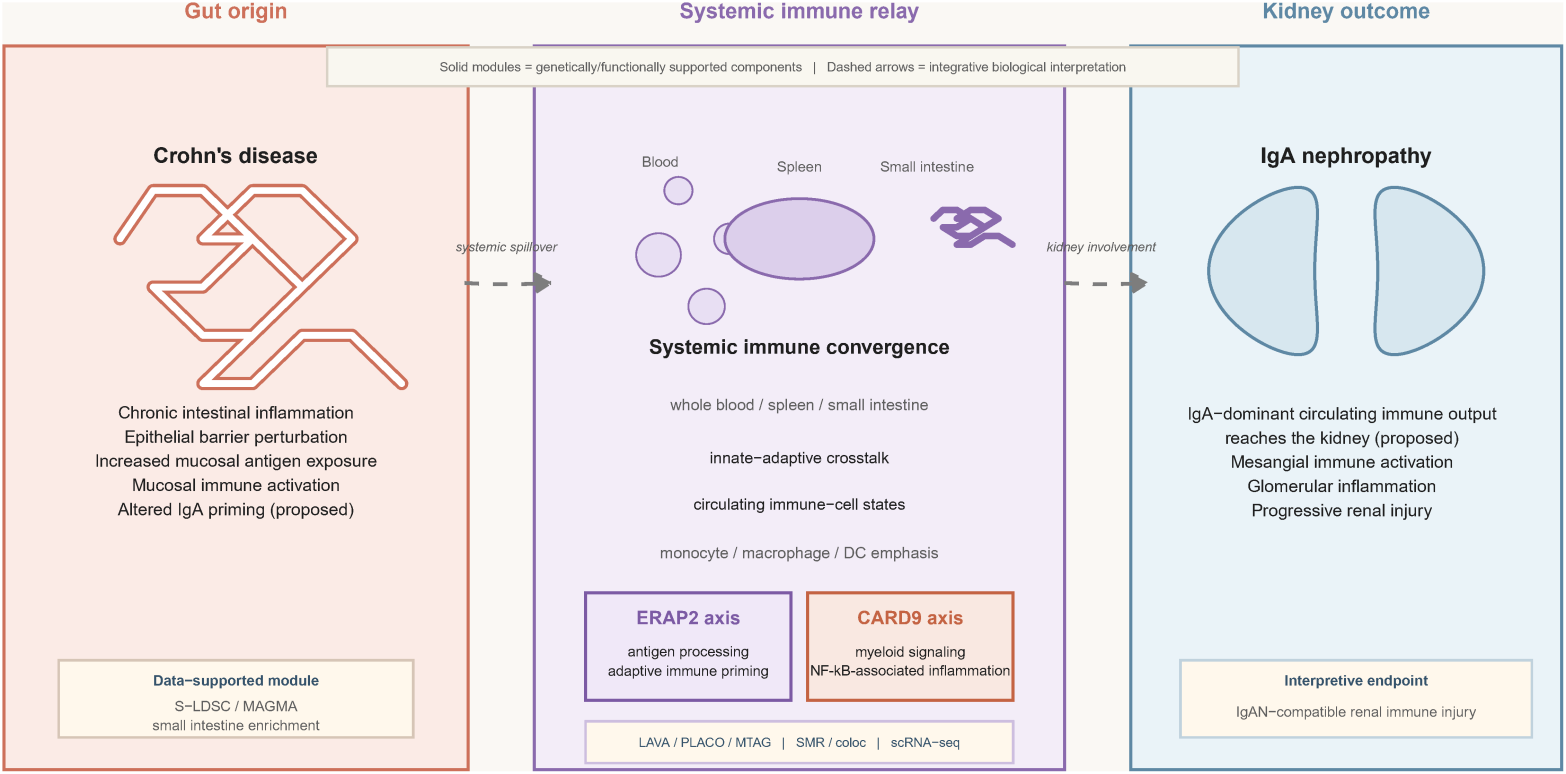
Proposed interpretive gut–immune–kidney framework for the Crohn’s disease–IgA nephropathy relationship. Schematic summary of the integrated findings from **Figures 1**-**5**. Solid modules denote genetically or functionally supported components, and dashed arrows denote biologically informed interpretation. The left panel summarizes the intestinal disease context in CD. The central panel highlights systemic immune convergence across whole blood, spleen, and small intestine, with *ERAP2* and *CARD9* prioritized as the two candidate functional axes linked to antigen processing/adaptive immune priming and myeloid/NF-κB-associated signaling, respectively. The right panel summarizes the proposed renal outcome context, including IgA-dominant circulating immune output, mesangial immune activation, glomerular inflammation, and progressive renal injury. This schematic represents a biologically informed interpretive framework rather than evidence of a demonstrated causal pathway. CD, Crohn’s disease; coloc, colocalization; DC, dendritic cell; IgAN, IgA nephropathy; LAVA, Local Analysis of [co]Variant Association; MAGMA, Multi-marker Analysis of GenoMic Annotation; MTAG, multi-trait analysis of GWAS; NF-κB, nuclear factor kappa B; PLACO, pleiotropic analysis under composite null hypothesis; S-LDSC, stratified linkage disequilibrium score regression; scRNA-seq, single-cell RNA sequencing; SMR, summary-data-based Mendelian randomization.

To synthesize the multilayered genetic and functional findings, we developed an integrative framework centered on a gut–immune–kidney axis (**Figure 6**). In this framework, CD-related chronic intestinal inflammation, epithelial barrier disruption, and increased mucosal antigen exposure represent plausible upstream processes within this framework. MR and GSMR analyses support a directional genetic association between CD liability and IgAN risk, while LDSC and GNOVA indicate that the two diseases share a broad polygenic background. Tissue and pathway enrichment analyses further localize this shared risk to systemic immune compartments—including whole blood, spleen, and small intestine—rather than kidney-intrinsic regulatory programs. *ERAP2* and *CARD9* were prioritized as candidate functional nodes, implicating antigen processing and presentation and myeloid NF-κB-associated inflammatory signaling, respectively, with their immune-cell context supported by single-cell analyses. Taken together, these findings are consistent with a framework in which CD-related mucosal immune dysregulation may contribute to IgAN-compatible glomerular injury through systemic immune amplification and IgA-skewed circulating immune output.

### 3.7 Clinical support for a preferential Crohn’s disease–IgA nephropathy association

To assess phenotypic support for the genetic findings at the clinical level, we analyzed a single-center hospitalized IBD cohort comprising 9,371 patients, including 7,149 with CD and 2,222 with UC, and compared renal outcomes between the two subtypes (**Supplementary Figure 4**; **Table 1**). Biopsy-proven IgAN was more frequent in CD than in UC [38/7,149 (0.53%) vs. 4/2,222 (0.18%)], and the difference remained significant in the Firth-adjusted analysis controlling for age and sex (OR = 3.40, 95% CI 1.28–11.28, P = 0.0125). For the secondary outcome, any biopsy-confirmed renal parenchymal disease was also more frequent in CD than in UC [82/7,149 (1.15%) vs. 14/2,222 (0.63%)], with a Firth-adjusted OR of 2.56 (95% CI 1.41–4.95, P = 0.0016). These clinical findings were consistent with the genetic analyses and further support a stronger association of IgAN with CD than with UC within the IBD spectrum.

**Table 1:** Retrospective clinical comparison of renal outcomes in Crohn’s disease and ulcerative colitis.

Outcome	CD, n/N (%)	UC, n/N (%)	RR (95% CI)	Crude OR (95% CI)	P value	Firth-adjusted OR (95% CI)	Adjusted P value
Biopsy-proven IgA nephropathy	38/7149 (0.53%)	4/2222 (0.18%)	2.95 (1.10–7.94)	2.96 (1.07–11.44)	**0.0286**	3.40 (1.28–11.28)	**0.0125**
Any biopsy-confirmed renal parenchymal disease	82/7149 (1.15%)	14/2222 (0.63%)	1.82 (1.04–3.18)	1.83 (1.03–3.50)	**0.0394**	2.56 (1.41–4.95)	**0.0016**

Data are presented as n/N (%), unless otherwise indicated.

The primary outcome was biopsy-proven IgA nephropathy, confirmed using kidney biopsy examination and diagnosis fields.

The secondary outcome was any biopsy-confirmed renal parenchymal disease, including IgA nephropathy and other histopathologically confirmed renal parenchymal lesions. Nephrolithiasis, renal dysfunction without pathological confirmation, and non-parenchymal or urological conditions were not included.

Risk ratios and corresponding 95% confidence intervals were estimated using the Koopman asymptotic score method. Crude odds ratios were estimated by conditional maximum likelihood, with exact 95% confidence intervals; crude P values were obtained using Fisher’s exact test.

Adjusted odds ratios were estimated using Firth bias-reduced logistic regression with adjustment for age and sex. Corresponding 95% confidence intervals were derived from the penalized profile likelihood, and adjusted P values were obtained using penalized likelihood ratio tests. CD, Crohn’s disease; UC, ulcerative colitis; RR, risk ratio; OR, odds ratio; CI, confidence interval.

Bold values indicate statistical significance at P < 0.05.

## 4 Discussion

In this study, we combined multilayered genetic analyses with phenotypic evidence from a retrospective clinical cohort and identified a CD–skewed association between IBD subtypes and IgAN. MR and GSMR supported a directional association between genetic liability to CD and increased risk of IgAN, whereas UC showed no consistent genetic effect. Genetic correlation analyses further revealed broad polygenic sharing between the two diseases, and functional enrichment pointed to systemic immune-related tissues rather than kidney-intrinsic mechanisms alone. Through local genetic architecture, colocalization, transcriptomic prioritization, and single-cell analyses, *ERAP2* and *CARD9* were prioritized as the leading candidate genes across evidence layers. In the clinical cohort, patients with CD showed higher frequencies of biopsy-confirmed IgAN and biopsy-confirmed renal parenchymal disease. Together, these findings support a shared immune-genetic framework connecting CD and IgAN and provide a biologically plausible basis for a proposed gut–immune–kidney axis.

This disease-subtype specificity is biologically informative. Previous clinical studies have linked IBD to IgAN, but whether this association reflects a generic effect of intestinal inflammation or subtype-specific disease biology has remained unclear (7, 8). Our findings do not support the simple interpretation that intestinal inflammation broadly increases the risk of IgAN. Instead, they suggest that immune features specific to CD may be more directly involved in IgAN susceptibility. Compared with UC, CD can involve the small intestine, particularly the ileum and ileocolonic regions, and is characterized by transmural inflammation, impaired intestinal barrier function, increased microbial antigen exposure, systemic immune activation, and a higher propensity for extraintestinal manifestations (1–3). These features provide a plausible route by which mucosal immune dysregulation may extend into systemic immune perturbation, consistent with the mucosal immune theory and gut–kidney crosstalk in IgAN pathogenesis (4, 5). By contrast, UC did not show consistent genetic evidence of association with IgAN in the available datasets, suggesting that this link more likely reflects CD–specific biology rather than a shared effect of IBD as a whole.

Beyond previously reported locus-level overlap between IBD and IgAN, genome-wide genetic correlation analyses revealed broad polygenic sharing between CD and IgAN, with consistently positive estimates of moderate magnitude, approximately in the range of 0.3–0.4, a level that falls within the range of meaningful cross-disease genetic correlations reported for immune-related traits (25). The concordant LDSC and GNOVA results suggest genome-wide shared susceptibility rather than signal overlap at a few pleiotropic loci (26, 27). This finding reframes the CD–IgAN link from a locus-level observation to a genome-wide shared genetic architecture. Viewed alongside large-scale GWAS showing immune-related risk pathways in both IgAN and IBD, this shared background likely points to a common immunogenetic basis (10, 11, 14, 15). By contrast, UC did not show genetic correlation of comparable magnitude with IgAN, further supporting that the shared architecture is skewed toward CD rather than representing a general effect of IBD.

This genome-wide sharing provides important context for interpreting the directional signal from MR analyses. Cross-ancestry two-sample MR was more consistent with a directional association between genetic liability to CD and increased risk of IgAN, whereas reverse MR did not show a consistent genetic effect of IgAN on CD across populations. GSMR yielded estimates in both directions, but the reverse analysis retained only 11 instruments compared with 84 in the forward analysis and was therefore less precise (19, 23). However, CAUSE did not clearly favor the causal model over the sharing model, suggesting that correlated pleiotropy and shared immunogenetic susceptibility may also contribute to the observed association (24). The CD–IgAN relationship is therefore best interpreted as shared polygenic immune susceptibility accompanied by a possible directional genetic association, rather than as a demonstrated one-way causal pathway.

The tissue-enrichment results provide an important clue to the shared mechanism between CD and IgAN. S-LDSC showed that genetic signals for both diseases were enriched in active regulatory regions, including enhancers and open chromatin, suggesting that shared risk variants may act primarily by modulating immune-related gene expression. Further LDSC-SEG and MAGMA analyses showed that the shared signals were enriched in immune-related tissues, particularly whole blood and spleen, rather than kidney tissue (28–30). This pattern is consistent with previous IgAN genetic studies in which disease-associated loci and prioritized genes converged on immune and inflammatory pathways, including blood and immune-cell–related signals (11). Although IgAN manifests clinically as glomerular injury, its upstream susceptibility may not be driven by kidney-intrinsic mechanisms alone. Instead, the kidney may represent the downstream target of mucosal and systemic immune dysregulation, with aberrant IgA production and circulating immune complexes linking immune activation to glomerular injury (4, 5, 42). Together, these findings suggest that the CD–IgAN link is more likely to involve systemic immune regulation and mucosal immune activation than a primarily kidney-origin shared genetic mechanism.

Building on the genome-wide shared genetic liability, local genetic architecture analyses further revealed regional heterogeneity in the shared signals between CD and IgAN. LAVA refined genetic correlation from the genome-wide level to specific genomic blocks, identifying several regions that jointly contributed to susceptibility to both diseases. Colocalization analyses then assessed whether each region was likely driven by a shared causal variant (34, 37). Prior statistical colocalization studies across related autoimmune diseases have shown that overlapping association signals within the same risk locus can arise from either shared or distinct causal variants (43). In line with this distinction, a positive local genetic correlation does not necessarily imply a single shared causal variant. Candidate gene prioritization therefore depended not on any single analytic result, but on convergence across local correlation, colocalization, transcriptional regulation, and functional annotation (34, 36, 37). The *ERAP2* and *CARD9* regions showed the most consistent cross-disease evidence across these layers and formed the focal points of our mechanistic interpretation. By contrast, several LAVA-positive regions lacked convincing colocalization support, suggesting they may contain nearby but distinct disease-specific variants rather than a shared causal signal. The negative local genetic correlation at chr18 adds a further layer of complexity, suggesting possible antagonistic effects or genetic heterogeneity between CD and IgAN at this locus; the biological basis of this finding remains unclear and will require larger GWAS and functional dissection to resolve.

*ERAP2* provides a plausible antigen-processing axis linking CD and IgAN. As a key regulator of peptide trimming and MHC class I antigen presentation, *ERAP2* may influence mucosal antigen processing in the intestine, thereby shaping local immune activation and contributing to downstream systemic IgA responses (44). In this study, the *ERAP2* region showed strong cross-disease colocalization and functional support across blood, ileal, and colonic tissues (45). However, this prioritization should be interpreted with caution: the evidence rests primarily on colocalization and biological plausibility, as the IgAN-side SMR analysis did not reach statistical significance. *ERAP2* should therefore be regarded as a mechanistically plausible candidate, whose role in IgAN remains to be confirmed through larger IgAN GWAS, context-relevant eQTL resources, and functional experiments (11).

*CARD9* may represent a myeloid innate immune bridge between CD and IgAN. As a key regulator of microbial sensing, antifungal and antibacterial immune responses, and innate immune signaling, *CARD9* biology aligns with key pathological features of CD, including gut microbial dysbiosis, impaired barrier function, and aberrant innate immune activation (46, 47). In this study, *CARD9* showed a strong whole-blood eQTL colocalization signal, and single-cell analysis further indicated that its expression was preferentially enriched in myeloid immune populations, including monocytes, macrophages, and dendritic cells (36, 37). Together, these findings suggest that CARD9-related myeloid immune responses may help translate microbial stimulation into systemic immune perturbation, pointing to myeloid innate immune activation as a key component of the shared disease biology between CD and IgAN.

Taken together, these findings are consistent with a biologically plausible gut–immune–kidney framework. Within this framework, CD-related mucosal immune activation and altered microbial antigen handling may contribute to systemic immune perturbation and increased susceptibility to IgA dysregulation and glomerular injury (4, 5, 42). *ERAP2* and *CARD9* may represent two complementary candidate components within this framework: antigen processing and presentation for *ERAP2*, and microbial sensing and myeloid innate immune activation for *CARD9*. This framework may help explain why the shared genetic signals are enriched primarily in systemic immune-related tissues while the clinical phenotype manifests as kidney injury, and may reconcile the apparent disconnect between the intestinal origin of genetic risk and the renal manifestation of IgAN (5, 6).

The clinical cohort provided phenotypic support for the genetic findings. Previous nationwide cohort studies and systematic reviews have documented a clinical link between IBD and IgAN, but whether this association differs by IBD subtype has remained uncertain (7, 8). In our cohort, patients with CD had a higher frequency of biopsy-confirmed IgAN than patients with UC, and biopsy-confirmed renal parenchymal disease was also more frequently recorded, consistent with the CD-skewed signal from the genetic analyses. These findings provide real-world phenotypic corroboration of the genetic results, not independent causal evidence. Renal manifestations in CD may be underrecognized in routine care (9). These observations support further evaluation of whether monitoring hematuria, proteinuria, and kidney function may improve the detection of renal involvement in patients with CD (41). Prospective studies are needed to determine whether CD activity, disease location, barrier dysfunction, or treatment exposure modifies the risk of IgAN.

We evaluated the CD–IgAN relationship through complementary genetic, functional, cellular, and clinical evidence rather than relying on a single analytic framework. Bidirectional MR analyses across European and East Asian populations further strengthened the cross-population robustness of the directional inference.

This study also has limitations. Although MR and GSMR supported a directional association between genetic liability to CD and IgAN risk, the CAUSE results indicated that shared genetic components may also contribute. Instrument-level heterogeneity in some MR analyses further suggests that the directional effect should be interpreted alongside the broad polygenic sharing between the two diseases. The available IgAN GWAS datasets remain limited in sample size and ancestral diversity, reducing power for some gene-level analyses. *ERAP2* did not reach significance in the IgAN-side SMR analysis, and its role will require validation in larger datasets and functional experiments. eQTL and tissue-reference resources remain limited by sample size, tissue source, and cellular composition, with intestinal and immune-cell-type-specific data particularly sparse. Single-cell analyses provided cellular context but cannot establish functional causality. Finally, the clinical cohort was single-center, hospital-based, and retrospective; biopsy-confirmed IgAN may be subject to ascertainment bias arising from differences in follow-up duration, kidney-biopsy rates, and renal testing frequency, while residual confounding related to disease severity and treatment exposure cannot be excluded. Whether these findings generalize beyond this setting will require multicenter prospective data.

## 5 Conclusions

The integrated genetic, functional, cellular, and clinical evidence supports shared immune-genetic architecture between CD and IgAN, with a possible directional genetic association from CD liability to IgAN risk. The enrichment of shared signals in systemic immune compartments, together with the prioritization of ERAP2 and CARD9, points to complementary roles for antigen processing and myeloid innate immune signaling within a proposed gut–immune–kidney framework. Larger multi-ancestry GWAS, context-specific functional studies, and prospective multicenter cohorts are needed to further clarify the underlying biology and clinical relevance.

## Data Availability

The publicly available GWAS, eQTL, and single-cell RNA-sequencing datasets analyzed in this study are available from the sources listed in **Supplementary Table 1**. De-identified clinical data may be made available by the corresponding authors upon reasonable request, subject to institutional and ethical approval.
