# Supplementary material for: Shared genetic architecture between Crohn’s disease and IgA nephropathy: implications for a gut–immune–kidney axis
Source: Front Immunol. 2026 Aug 4;17:1905767. doi: 10.3389/fimmu.2026.1905767 (PMC13481795; doi:10.3389/fimmu.2026.1905767)
Supplement: Supplementary file 1 [file DataSheet1.pdf]

STROBE-MR checklist

Manuscript title: Shared Genetic Architecture Between Crohn's Disease and IgA Nephropathy: Implications for a Gut–Immune–Kidney Axis

Reporting guideline: Strengthening the Reporting of Observational Studies in Epidemiology using Mendelian Randomization (STROBE-MR).

Item No.	Section	Checklist item	Manuscript location
1	TITLE and ABSTRACT	Indicate Mendelian randomization (MR) as the study's design in the title and/or the abstract if that is a main purpose of the study	Abstract (Methods); Keywords
**INTRODUCTION**			
2	Background	Explain the scientific background and rationale for the reported study. What is the exposure? Is a potential causal relationship between exposure and outcome plausible? Justify why MR is a helpful method to address the study question	Introduction
3	Objectives	State specific objectives clearly, including pre-specified causal hypotheses (if any). State that MR is a method that, under specific assumptions, intends to estimate causal effects	Introduction; Methods 2.2
**METHODS**			
4	Study design and data sources	Present key elements of the study design early in the article. Consider including a table listing sources of data for all phases of the study. For each data source contributing to the analysis, describe the following:	Methods 2.1–2.2; Supplementary Table S1
	a)	Setting: Describe the study design and the underlying population, if possible. Describe the setting, locations, and relevant dates, including periods of recruitment, exposure, follow-up, and data collection, when available.	Methods 2.1-2.2; Supplementary Table S1
	b)	Participants: Give the eligibility criteria, and the sources and methods of selection of participants. Report the sample size, and whether any power or sample size calculations were carried out prior to the main analysis	Methods 2.1-2.2; Supplementary Table S1; Methods 2.9; Supplementary Figure S4; Table 1
	c)	Describe measurement, quality control and selection of genetic variants	Methods 2.2; Supplementary Methods 2.2
	d)	For each exposure, outcome, and other relevant variables, describe methods of assessment and diagnostic criteria for diseases	Methods 2.1-2.2; Supplementary Table S1; Methods 2.9; Supplementary Table S5
	e)	Provide details of ethics committee approval and participant informed consent, if relevant	Methods 2.1; Methods 2.9; Ethics approval and consent to participate
5	Assumptions	Explicitly state the three core IV assumptions for the main analysis (relevance, independence and exclusion restriction) as well assumptions for any additional or sensitivity analysis	Methods 2.2; Supplementary Methods 2.2; Discussion
6	Statistical methods: main analysis	Describe statistical methods and statistics used	Methods 2.2; Supplementary Methods 2.2
	a)	Describe how quantitative variables were handled in the analyses (i.e., scale, units, model)	Methods 2.2; Results 3.1; Supplementary Table S2
	b)	Describe how genetic variants were handled in the analyses and, if applicable, how their weights were selected	Methods 2.2; Supplementary Methods 2.2
	c)	Describe the MR estimator (e.g. two-stage least squares, Wald ratio) and related statistics. Detail the included covariates and, in case of two-sample MR, whether the same covariate set was used for adjustment in the two samples	Methods 2.2; Supplementary Methods 2.2
	d)	Explain how missing data were addressed	Not applicable to individual-level missing data;

Item No.	Section	Checklist item	Manuscript location
			analyses used published GWAS summary statistics. Instrument handling is described in Methods 2.2 and Supplementary Methods 2.2.
	e)	If applicable, indicate how multiple testing was addressed	Methods 2.2; Supplementary Methods 2.2; Results 3.1; Supplementary Table S2
7	Assessment of assumptions	Describe any methods or prior knowledge used to assess the assumptions or justify their validity	Methods 2.2; Supplementary Methods 2.2; Results 3.1; Supplementary Table S2; Supplementary Figure S1
8	Sensitivity analyses and additional analyses	Describe any sensitivity analyses or additional analyses performed (e.g. comparison of effect estimates from different approaches, independent replication, bias analytic techniques, validation of instruments, simulations)	Methods 2.2; Supplementary Methods 2.2; Results 3.1; Supplementary Table S2; Supplementary Figure S1
9	Software and pre-registration		
	a)	Name statistical software and package(s), including version and settings used	Methods 2.10; Supplementary Methods 2.10
	b)	State whether the study protocol and details were pre-registered (as well as when and where)	Not pre-registered; no trial registration was applicable.
**RESULTS**			
10	Descriptive data		
	a)	Report the numbers of individuals at each stage of included studies and reasons for exclusion. Consider use of a flow diagram	Methods 2.1; Supplementary Table S1; Methods 2.9; Supplementary Figure S4; Table 1
	b)	Report summary statistics for phenotypic exposure(s), outcome(s), and other relevant variables (e.g. means, SDs, proportions)	Supplementary Table S1; Table 1
	c)	If the data sources include meta-analyses of previous studies, provide the assessments of heterogeneity across these studies	Supplementary Table S1; original GWAS studies cited in References
	d)	For two-sample MR: Provide justification of the similarity of the genetic variant-exposure associations between the exposure and outcome samples Provide information on the number of individuals who overlap between the exposure and outcome studies	Methods 2.1-2.3; Supplementary Table S1; LDSC cross-trait intercept analyses in Methods 2.3
11	Main results		
	a)	Report the associations between genetic variant and exposure, and between genetic variant and outcome, preferably on an interpretable scale	GWAS summary statistics described in Methods 2.1 and Supplementary Table S1; MR estimates in Results 3.1 and Supplementary Table S2
	b)	Report MR estimates of the relationship between exposure and outcome, and the measures of uncertainty from the MR analysis, on an interpretable scale, such as odds ratio or relative risk per SD difference	Results 3.1; Figure 1A-B; Supplementary Table S2
	c)	If relevant, consider translating estimates of relative risk into absolute risk for a meaningful time period	Results 3.7; Table 1; MR estimates are reported as ORs/effect estimates rather than absolute risks
	d)	Consider plots to visualize results (e.g. forest plot, scatterplot of associations between genetic variants and outcome versus between genetic variants and exposure)	Figure 1A-B; Supplementary Figure S1
12	Assessment of assumptions		
	a)	Report the assessment of the validity of the assumptions	Results 3.1; Supplementary Table S2; Supplementary Figure S1; Discussion
	b)	Report any additional statistics (e.g., assessments of heterogeneity across genetic variants, such as I2, Q statistic or E-value)	Methods 2.2; Supplementary Methods 2.2; Supplementary Table S2
13	Sensitivity analyses and additional analyses		
	a)	Report any sensitivity analyses to assess the robustnes s of the main results to violations of the	Methods 2.2; Supplementary Methods 2.2; Results 3.1; Supplementary Table S2; Supplementary Figure S1

Item No.	Section	Checklist item	Manuscript location
		assumptions	
	b)	Report results from other sensitivity analyses or additional analyses	Results 3.1; Figure 1B; Supplementary Table S2; Supplementary Figure S1
	c)	Report any assessment of direction of causal relationship (e.g., bidirectional MR)	Methods 2.2; Supplementary Methods 2.2; Results 3.1; Figure 1A-B; Supplementary Table S2
	d)	When relevant, report and compare with estimates from non-MR analyses	Results 3.2; Figure 1C-D; Results 3.7; Table 1
	e)	Consider additional plots to visualize results (e.g., leave-one-out analyses)	Figure 1A-B; Supplementary Figure S1
**DISCUSSION**			
14	Key results	Summarize key results with reference to study objectives	Discussion, first paragraph
15	Limitations	Discuss limitations of the study, taking into account the validity of the IV assumptions, other sources of potential bias, and imprecision. Discuss both direction and magnitude of any potential bias and any efforts to address them	Discussion, limitations paragraph
16	Interpretation		
	a)	Meaning: Give a cautious overall interpretation of results in the context of their limitations and in comparison with other studies	Discussion
	b)	Mechanism: Discuss underlying biological mechanisms that could drive a potential causal relationship between the investigated exposure and the outcome, and whether the gene-environment equivalence assumption is reasonable. Use causal language carefully, clarifying that IV estimates may provide causal effects only under certain assumptions	Discussion; Figure 6
	c)	Clinical relevance: Discuss whether the results have clinical or public policy relevance, and to what extent they inform effect sizes of possible interventions	Discussion; Results 3.7; Table 1
17	Generalizability	Discuss the generalizability of the study results (a) to other populations, (b) across other exposure periods/timings, and (c) across other levels of exposure	Discussion, limitations paragraph; Methods 2.1
**OTHER INFORMATION**			
18	Funding	Describe sources of funding and the role of funders in the present study and, if applicable, sources of funding for the databases and original study or studies on which the present study is based	Funding
19	Data and data sharing	Provide the data used to perform all analyses or report where and how the data can be accessed, and reference these sources in the article. Provide the statistical code needed to reproduce the results in the article, or report whether the code is publicly accessible and if so, where	Data availability statement generated during submission; Methods 2.1; Supplementary Table S1
20	Conflicts of Interest	All authors should declare all potential conflicts of interest	Conflict-of-interest declaration generated during submission

Checklist references: Skrivankova et al., STROBE-MR: guidelines for strengthening the reporting of Mendelian randomization studies.
