# Supplementary material for: Shared genetic architecture between Crohn’s disease and IgA nephropathy: implications for a gut–immune–kidney axis
Source: Front Immunol. 2026 Aug 4;17:1905767. doi: 10.3389/fimmu.2026.1905767 (PMC13481795; doi:10.3389/fimmu.2026.1905767)
Supplement: Supplementary file 2 [file DataSheet2.pdf]

Supplementary Materials

Contents

Section / item	Printed page(s)
Supplementary Methods	1-6
Supplementary Figure Legends	7-8
Supplementary Figure S1. CAUSE sensitivity analysis for CD-IgAN causal versus shared-effect models	9
Supplementary Figure S2. Extended tissue-resolved colocalization support for ERAP2 and CARD9	10
Supplementary Figure S3. Alternative t-SNE visualization of the single-cell analysis	11
Supplementary Figure S4. Study flow in the retrospective clinical cohort	12
Supplementary Table S1. Summary of datasets and sample characteristics used in the study	13
Supplementary Table S2. Bidirectional two-sample MR, instrument characteristics, sensitivity statistics, and GSMR results	14-15
Supplementary Table S3. Local genetic architecture and cross-trait locus evidence from LAVA, PLACO, and MTAG	16
Supplementary Table S4. Regional colocalization, SMR effect estimates, and gene-level results at the ERAP2 and CARD9 loci	17
Supplementary Table S5. Operational definitions of exposure, renal outcomes, and covariates	18
Supplementary Table S6. Complete MAGMA gene-set results for Crohn's disease and IgA nephropathy	Separate Excel file

Page numbers refer to the printed page numbers in this supplementary PDF. Supplementary Table S6 is provided separately as an Excel file.

Supplementary Methods

Abbreviations: CD, Crohn's disease; UC, ulcerative colitis; IBD, inflammatory bowel disease; IgAN, IgA nephropathy; GWAS, genome-wide association study; eQTL, expression quantitative trait locus; PBMC, peripheral blood mononuclear cells; RNA, ribonucleic acid; FASTQ, FASTA with quality scores; GRCh38, Genome Reference Consortium Human Build 38; UMI, unique molecular identifier; PCA, principal component analysis; NK, natural killer; UMAP, Uniform Manifold Approximation and Projection; t-SNE, t-distributed stochastic neighbor embedding; SNP, single nucleotide polymorphism; MR, Mendelian randomization; GSMR, generalized summary-data-based Mendelian randomization; CAUSE, causal analysis using summary effect estimates; IVW, inverse-variance weighted; LDSC, linkage disequilibrium score regression; GNOVA, genetic covariance analyzer; S-LDSC, stratified linkage disequilibrium score regression; LDSC-SEG, linkage disequilibrium score regression for specifically expressed genes; GTEx, Genotype-Tissue Expression; PLACO, pleiotropic analysis under a composite null hypothesis; MAGMA, Multi-marker Analysis of GenoMic Annotation; MTAG, multi-trait analysis of genome-wide association studies; MR-PRESSO, Mendelian Randomization Pleiotropy RESidual Sum and Outlier; LAVA, Local Analysis of [co]Variant Association; SMR, summary-data-based Mendelian randomization; FDR, false discovery rate; PPH4, posterior probability for hypothesis 4; LD, linkage disequilibrium; HEIDI, heterogeneity in dependent instruments.

2.2 Bidirectional Mendelian Randomization, GSMR, and CAUSE

Instrumental variables were selected at the genome-wide significance threshold ($P < 5 \times 10^{-8}$) and subjected to LD clumping using an r^2 threshold of 0.001 within a 10,000-kb window. Exposure and outcome variants were harmonized according to the effect alleles; ambiguous palindromic variants were excluded, and no proxy variants were used. Instrument strength was evaluated using the F statistic, with variants having $F \leqslant 10$ excluded. Cumulative R^2 was calculated for the retained instruments, and Steiger directionality testing was used to assess whether each variant explained more variance in the exposure than in the outcome. Instrument counts before clumping, after LD clumping, after harmonization, after MR-PRESSO outlier removal, and in the final analyses, together with the minimum and mean F statistics, cumulative R^2, and Steiger results, are reported in Supplementary Table S2.

IVW served as the primary estimator. A random-effects model was used when Cochran's Q test indicated heterogeneity; otherwise, a fixed-effects model was applied. (20) MR-Egger, weighted median, weighted mode, and simple mode analyses were performed as sensitivity analyses. Heterogeneity was assessed using Cochran's Q test, and directional horizontal pleiotropy was evaluated using the MR-Egger intercept test. (21) MR-PRESSO was used to identify outlier instruments; where outliers were detected, the analyses were rerun after their removal, and the corrected estimates were retained as the final results. (22) For the primary IVW analyses, Benjamini–Hochberg FDR correction was applied separately to the six forward and six reverse comparisons

across both ancestries and the three IBD phenotypes. Sensitivity estimators were used to assess robustness and were not treated as separate primary hypothesis tests. Complete MR estimates and diagnostic statistics are provided in Supplementary Table S2.

For the CD–IgAN pair, GSMR was performed bidirectionally, with the HEIDI-outlier procedure used to remove variants showing evidence of horizontal pleiotropy. (23) The number of retained instruments was reported separately for each direction and was considered when interpreting differences in statistical power between the forward and reverse analyses. CAUSE was additionally used to compare the causal and sharing models and to assess whether the observed association was more consistent with a directional effect or correlated horizontal pleiotropy arising from shared genetic architecture. (24)

2.3 Genome-wide genetic correlation analyses: LDSC and GNOVA

For LDSC, we used European reference LD scores to estimate SNP heritability and pairwise genetic correlation, together with standard errors and P values. (25) Both the standard intercept model and the constrained no-intercept model were run. The cross-trait intercept from the standard model was used to assess potential sample overlap between GWAS datasets, whereas the constrained model provided a complementary estimate under the assumption of minimal sample overlap. (26)

GNOVA was then applied to estimate genetic covariance and genetic correlation between trait pairs. Results were reported both before and after correction to assess robustness. (27)

2.4 Functional enrichment and tissue/pathway analyses

S-LDSC was performed using the baseline-LD v2.2 annotation set to assess heritability enrichment across functional annotation categories. (28) For each category, we estimated the proportion of heritability explained, the corresponding enrichment, and the associated P value. Multiple testing was controlled separately for CD and IgAN across all tested annotations with valid P values using the Benjamini–Hochberg procedure, with FDR < 0.05 considered statistically significant.

Tissue-specific enrichment was assessed using both LDSC-SEG and MAGMA. LDSC-SEG used tissue-specific expression profiles from 53 GTEx tissues to test whether disease-associated genetic signals were enriched in specific tissues. (29) MAGMA used gene-level association statistics together with GTEx tissue-expression data for gene-property analysis. (30) Both methods were applied across all available tissues. Multiple testing was controlled separately within each trait and analytical method across all tested tissues using the Benjamini–Hochberg procedure, with FDR < 0.05 considered statistically significant.

MAGMA was also used for competitive gene-set analysis in CD and IgAN using the Molecular Signatures Database human gene-set collection, release 2023.1. All eligible gene sets were tested separately for each trait, and multiple testing was controlled across all tested gene sets within each trait using the Benjamini–Hochberg procedure. An FDR < 0.05 was considered statistically significant.

2.5 Cross-trait locus discovery: PLACO and MTAG

MTAG was performed using standardized GWAS summary statistics, including SNP identifiers,

alleles, effect sizes, standard errors, P values, allele frequencies, sample sizes, and Z scores. Variants with a minor allele frequency > 0.01 were retained. Potential sample overlap was accommodated by estimating, rather than constraining, the error covariance matrix. Trait-specific mean χ^2 values, genetic and error covariance matrices, GWAS-equivalent sample sizes, and maximum FDR values were obtained as diagnostic measures. MTAG generated trait-specific association estimates, and MTAG-enhanced associations were not interpreted as shared loci in isolation. (32)

PLACO was used to identify significant cross-trait signals using a Bonferroni-corrected threshold of $P < 6.80 \times 10^{-9}$, corresponding to 0.05 divided by the 7,352,033 tested variants. Z-score filtering and decorrelation were applied before PLACO testing. The correlation between the trait-specific Z statistics was estimated and used in the decorrelation procedure, thereby accounting for correlation potentially arising from sample overlap. (31)

Potential additional shared loci were identified in three steps. First, variants meeting the PLACO significance threshold were extracted and merged with the MTAG results. MTAG results were used to characterize the trait-specific association evidence but were not used alone to define shared loci. These variants were then subjected to LD clumping using the European 1000 Genomes reference panel with a 500-kb window and $r^2 < 0.3$ to obtain an initial set of LD-clumped lead signals. (33) Second, within this set, variants that reached genome-wide significance in both the original CD and IgAN GWAS were defined as known shared loci. These loci were subsequently evaluated using an expanded 1,000-kb window and $r^2 < 0.3$ to identify variants within their LD range. Third, variants in LD with these known shared loci were removed from the LD-clumped signal set. The remaining lead variants that were significant in PLACO but did not reach genome-wide significance in at least one of the original GWAS were retained as potential additional shared loci.

2.6 Local genetic architecture analysis: LAVA

LAVA analysis was based on European GWAS summary statistics and used the European 1000 Genomes reference panel together with the predefined semi-independent LD block file provided by LAVA (blocks_s2500_m25_f1_w200, GRCh37/hg19 version), which was derived from the LD block framework of Berisa and Pickrell (2016). (35)

Before running LAVA, we used LDSC to estimate the global genetic correlation between CD and IgAN and constructed a sample overlap covariance matrix from the LDSC output to account for potential sample overlap. (26)

LAVA was implemented in a two-step framework. First, univariate local heritability tests were performed within each LD block. Bivariate local genetic correlation analysis was then carried out only for blocks in which both traits passed the prespecified screening threshold (univariate $P < 0.05$). This threshold was used only as an entry criterion for the bivariate step and was not treated as a multiple-testing correction threshold. For blocks passing this screening step, local genetic correlation coefficients and corresponding significance levels were estimated. Statistical significance for bivariate results was determined using a Bonferroni-corrected threshold. Local genetic correlation results were subsequently used to nominate candidate regions for colocalization analysis.

2.7 Colocalization and summary-data-based Mendelian randomization

SMR analyses were carried out through the SMR Portal. Prior to portal submission, CD and IgAN GWAS loci were prescreened based on genome-wide significance ($P < 5 \times 10^{-8}$). The HEIDI test was applied to assess whether the SMR association was compatible with a shared variant rather than heterogeneity arising from linkage disequilibrium; HEIDI $P > 0.05$ was considered evidence against substantial heterogeneity. (36)

Candidate regions for colocalization were selected based on significant local genetic correlations identified by LAVA and signals prioritized by SMR. GWAS–GWAS and GWAS–eQTL colocalization analyses were performed. The GWAS–eQTL analyses used eQTLGen whole-blood data and GTEx v8 data from the spleen, terminal ileum, transverse colon, and sigmoid colon, where available. Each regional analysis covered a 1-Mb window on either side of the candidate gene. The coloc five-hypothesis framework was applied using prior probabilities of $p1 = 1 \times 10^{-4}$, $p2 = 1 \times 10^{-4}$, and $p12 = 1 \times 10^{-5}$. PPH4 ≥ 0.80 was considered strong support for colocalization. (37)

2.8 Single-cell RNA sequencing analysis

The nine PBMC samples from GSE134809 were analyzed using the deposited 10x Genomics count matrices generated against the GRCh38 reference genome; raw FASTQ files were not reprocessed, and no additional demultiplexing or alignment was performed. The matrices were imported into Seurat using Read10X and CreateSeuratObject, retaining genes detected in at least three cells and cells with at least 200 detected genes. Cells with more than 3,000 detected genes, more than 15,000 UMIs, mitochondrial RNA content >15%, or ribosomal RNA content >60% were excluded. Ambient RNA contamination was estimated using decontX, and cells with estimated contamination ⩾0.30 were removed. Doublets were identified separately for each sample using scDblFinder, and only singlets were retained for downstream analysis. (38)

After doublet removal, the data were re-normalized using NormalizeData with the LogNormalize method, followed by variable-feature selection, scaling, PCA, and Harmony correction for sample-level batch effects. Graph-based clustering was performed using FindNeighbors and FindClusters. Initial clustering based on the first 16 Harmony dimensions at a resolution of 0.02 yielded seven clusters, from which clusters expressing immune-lineage markers were retained for secondary analysis. These cells were subsequently reclustered using the first 20 Harmony dimensions at a resolution of 0.4, yielding 12 clusters that were consolidated into eight final populations: T cells, $CD8^{+}$ cytotoxic T cells, B cells, plasma cells, monocytes, macrophages, NK cells, and dendritic cells. Manual annotations were assigned de novo according to cluster-specific expression patterns and established canonical immune-lineage markers, including CD3D/CD3E for T cells, CD19/CD79A/MS4A1 for B cells, GNLY/NKG7/KLRD1 for NK cells, CD14 for monocytes, and CD1C/FCER1A for dendritic cells. UMAP and t-SNE embeddings were generated using RunUMAP and RunTSNE, respectively. (39)

2.9 Retrospective clinical cohort

Patients were included if they had a definite diagnosis of CD or UC during hospitalization, with

sufficient documentation to support IBD subtype classification. (40) Patients with ambiguous or provisional diagnoses were excluded, as were those in whom IgAN preceded the IBD diagnosis, to avoid bias from reversed temporal ordering. All patients in the final cohort had a confirmed IBD diagnosis before renal outcome ascertainment.

The primary outcome was biopsy-proven IgAN, and the secondary outcome was any biopsy-confirmed renal parenchymal disease. Both outcomes were ascertained from kidney biopsy records and the corresponding diagnostic documentation. Group differences were assessed using Fisher's exact test. Risk ratios were calculated with Koopman score 95% confidence intervals, and crude odds ratios with conditional exact 95% confidence intervals. Adjusted odds ratios were estimated using Firth bias-reduced logistic regression with profile-likelihood 95% confidence intervals, adjusting for age and sex.

2.10 Software and statistical environment

Unless otherwise specified, analyses were performed in R. For GSE134809, the publicly available count matrices, rather than raw FASTQ files, were used for downstream analysis. According to the original dataset metadata, the raw sequencing data had been processed by the data generators using Cell Ranger (v2.0) with the GRCh38 human reference genome. Downstream single-cell RNA-sequencing analyses were performed in R version 4.4.1 using the Seurat (v5.4.0) and SeuratObject (v5.3.0) R packages for count-matrix processing, quality control, normalization, dimensionality reduction, clustering, and visualization; the harmony R package (v1.2.4) for sample-level batch correction; the scDblFinder R package (v1.20.2) for doublet detection; and the decontX (v1.4.1) and celda (v1.22.1) R packages for ambient RNA contamination estimation.

All other R-based analyses were performed in R version 4.5.2. Two-sample Mendelian randomization and allele harmonization were conducted using the TwoSampleMR R package (v0.6.6). Horizontal pleiotropic outliers were detected and removed using the MRPRESSO R package (v1.0), and supplementary Mendelian randomization analyses were performed using the MendelianRandomization R package (v0.10.0). GSMR and HEIDI-outlier analyses were performed using the gsmr R package (GSMR2; v1.1.1), CAUSE analyses using the cause R package (v1.2.0.0335), colocalization analyses using the coloc R package (v5.2.3), and local genetic-correlation analyses using the LAVA R package (v0.1.5). Firth bias-reduced logistic regression was performed using the logistf R package (v1.26.1), and Koopman score confidence intervals for risk ratios were calculated using the DescTools R package (v0.99.60).

LDSC-based analyses, including LDSC, S-LDSC, and LDSC-SEG, were performed using the original LDSC command-line software (v1.0.1) in Python 2.7. GNOVA analyses were conducted using the GNOVA-2.0 command-line implementation, and MTAG analyses were conducted using the original MTAG command-line implementation (v1.0.8), in separately configured Python 2.7 environments. Gene-level, tissue-specific, and gene-set enrichment analyses were performed using the standalone MAGMA software (v1.10). PLACO analyses were performed using the original PLACO R script (v0.2.0). SMR and HEIDI analyses were conducted through the SMR Portal (accessed April 2,

2026).

Supplementary Figure Legends

Supplementary Figure S1. CAUSE sensitivity analysis for CD–IgAN causal versus shared-effect models.

(A) Upper panel summarizing CAUSE model-comparison statistics and posterior parameter distributions for the sharing and causal models. Tables report pairwise comparisons of the null, sharing, and causal models based on ELPD, together with posterior summaries of model parameters. Density plots show prior and posterior distributions of the key CAUSE parameters under the two models.

(B) Lower panel showing variant-level visualization under the sharing and causal models, together with ELPD contribution. Points represent variants plotted by their estimated effects on the two traits; point size reflects statistical significance, color indicates posterior assignment or model contribution, and diagonal reference lines denote model parameterization. CAUSE did not show clear superiority of the causal model over the sharing model and was therefore interpreted as a sensitivity analysis rather than a primary line of evidence.

Abbreviations: CAUSE, causal analysis using summary effect estimates; CD, Crohn's disease; ELPD, expected log pointwise posterior density; IgAN, IgA nephropathy.

Supplementary Figure S2. Extended tissue-resolved colocalization support for ERAP2 and CARD9.

Heatmap summarizing tissue-resolved colocalization support for ERAP2 and CARD9 across the analyzed CD and IgAN comparisons. Cell values denote PPH4 (%), and blank cells indicate tissue–disease pairs not analyzed. This supplementary panel extends the tissue-level colocalization summary shown in the main text and is restricted to the two genes retained in the final evidence framework.

Abbreviations: CD, Crohn's disease; IgAN, IgA nephropathy; PPH4, posterior probability for hypothesis 4.

Supplementary Figure S3. Alternative t-SNE visualization of the single-cell analysis.

(A) t-SNE embedding of the curated immune-cell atlas derived from PBMC scRNA-seq data from patients with CD. Retained immune subsets are annotated using the same cell-state framework as in the main-text UMAP analysis. **(B)** Density-based feature visualization of ERAP2 expression in the t-SNE embedding,

showing broad distribution across immune compartments. **(C)** Density-based feature visualization of CARD9 expression in the same embedding, showing enrichment in myeloid-associated compartments. The same annotated cells are shown using t-SNE as an alternative two-dimensional embedding.

Abbreviations: CD, Crohn's disease; PBMC, peripheral blood mononuclear cell; scRNA-seq, single-cell RNA sequencing; t-SNE, t-distributed stochastic neighbor embedding; UMAP, uniform manifold approximation and projection.

Supplementary Figure S4. Study flow in the retrospective clinical cohort.

Flowchart of the single-center hospitalized inflammatory bowel disease cohort from The Sixth Affiliated Hospital, Sun Yat-sen University (2012-10-01 to 2025-10-31). Hospitalized records were identified using discharge diagnosis codes for Crohn's disease (K50) or ulcerative colitis (K51), yielding the final analyzed cohort. The flowchart summarizes the distribution of any biopsy-confirmed renal parenchymal disease and biopsy-proven IgA nephropathy in Crohn's disease and ulcerative colitis. Renal parenchymal disease and biopsy-proven IgA nephropathy were confirmed using kidney biopsy examination and diagnosis fields.

A

	model1	model2	delta_elpd	se_delta_elpd	z	p
1	null	sharing	−3.3	2.50	−1.30	0.095
2	null	causal	−2.2	2.70	−0.82	0.210
3	sharing	causal	1.1	0.52	2.10	0.980

model	gamma	eta	q
Sharing	NA	0.49 (0.1, 0.77)	0.15 (0.03, 0.33)
Causal	0.01 (−0.06, 0.08)	0.48 (−0.19, 0.77)	0.14 (0.02, 0.35)

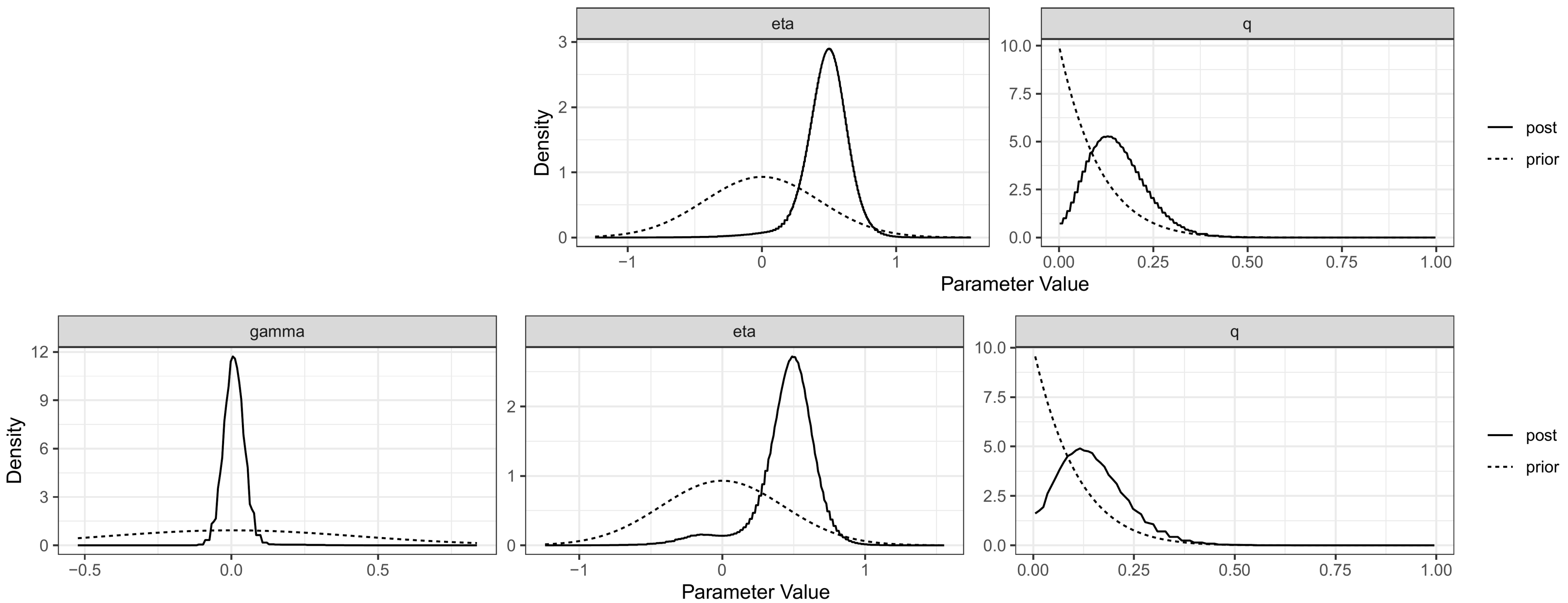

B

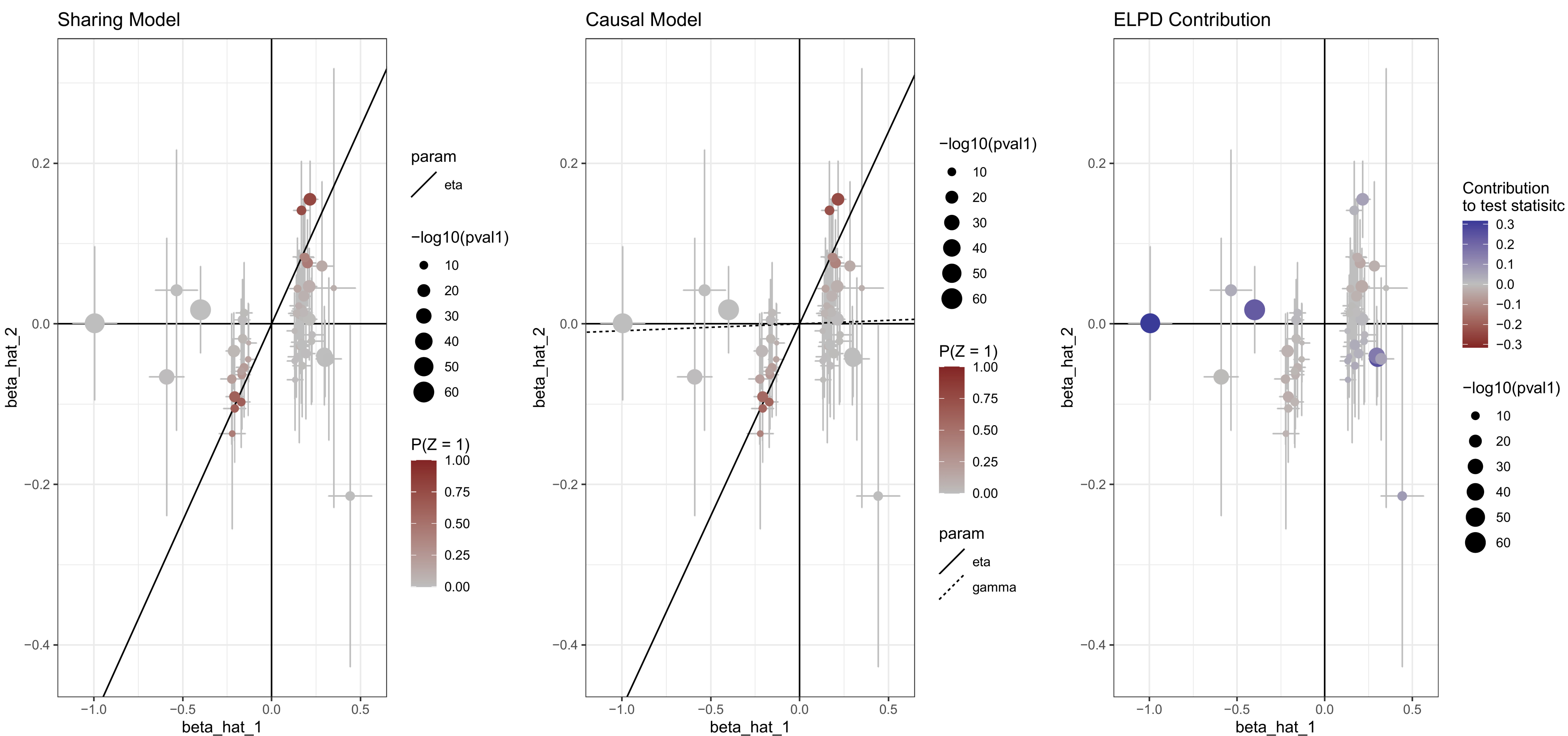

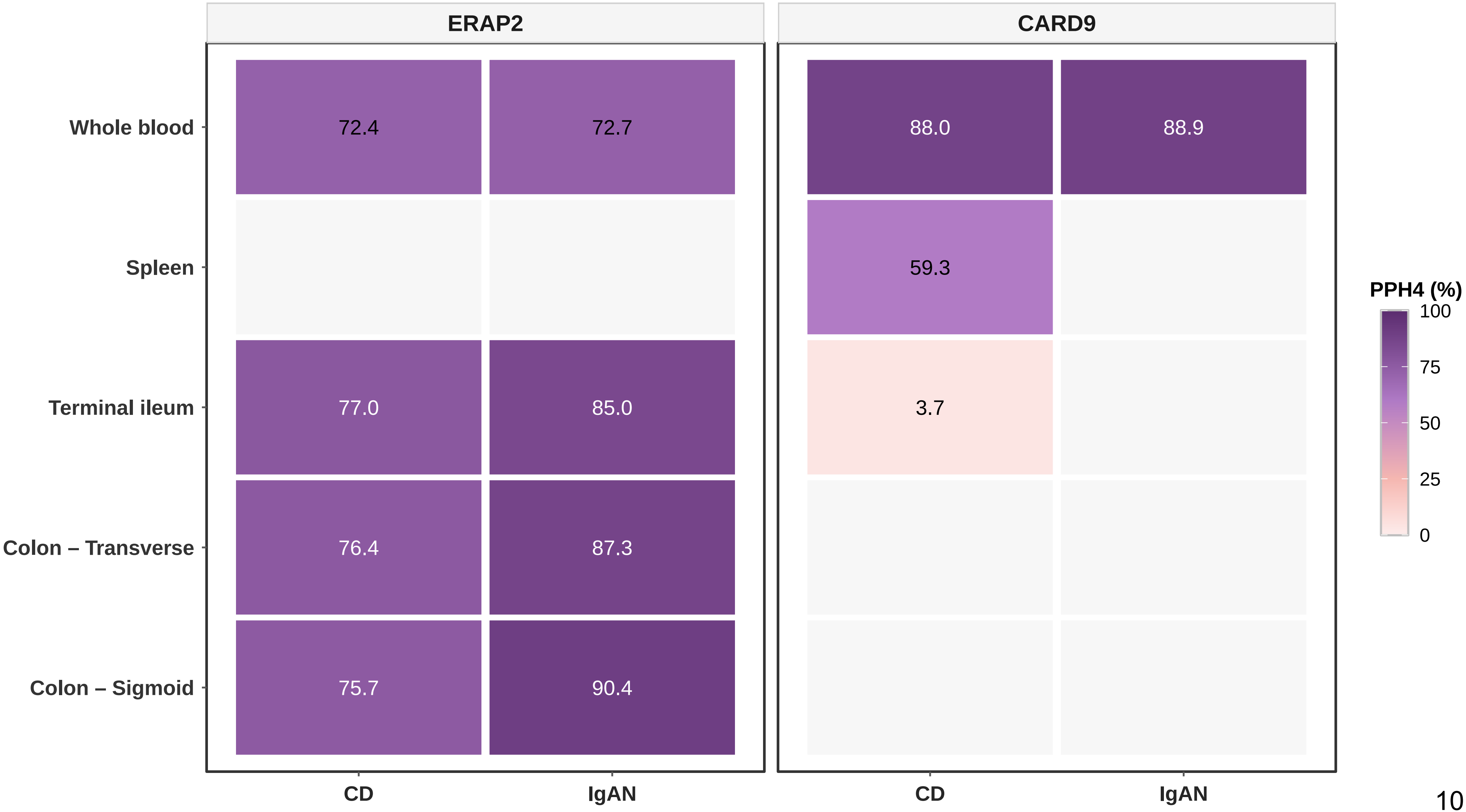

This supplementary heatmap extends the tissue−level colocalization summary shown in the main text. Blank cells indicate tissue−disease pairs not analyzed.

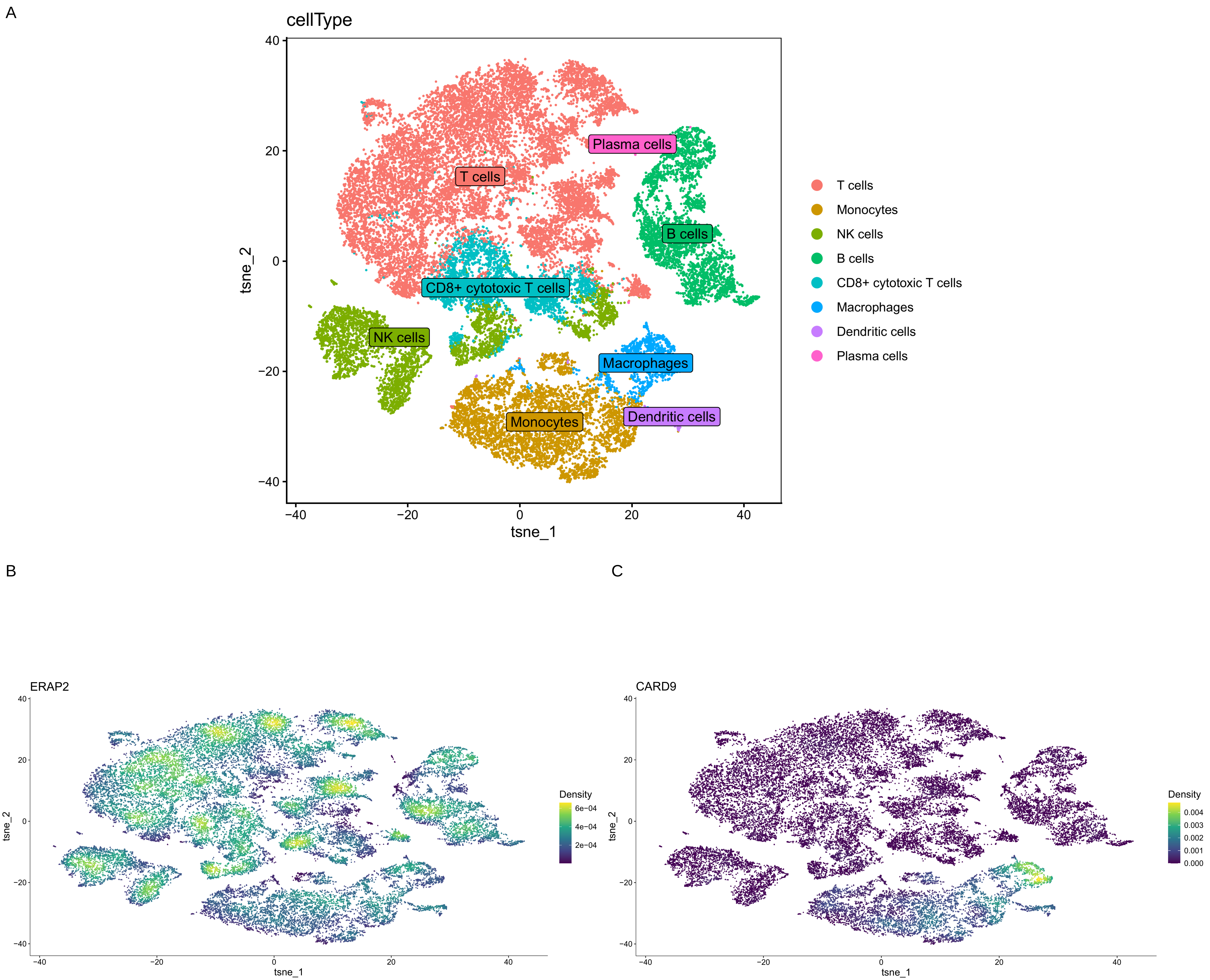

The same annotated cells are shown using t-SNE as an alternative two-dimensional embedding.

Study flow and renal outcome ascertainment

Single-center hospitalized inflammatory bowel disease cohort

The Sixth Affiliated Hospital, Sun Yat-sen University | 2012-10-01 to 2025-10-31

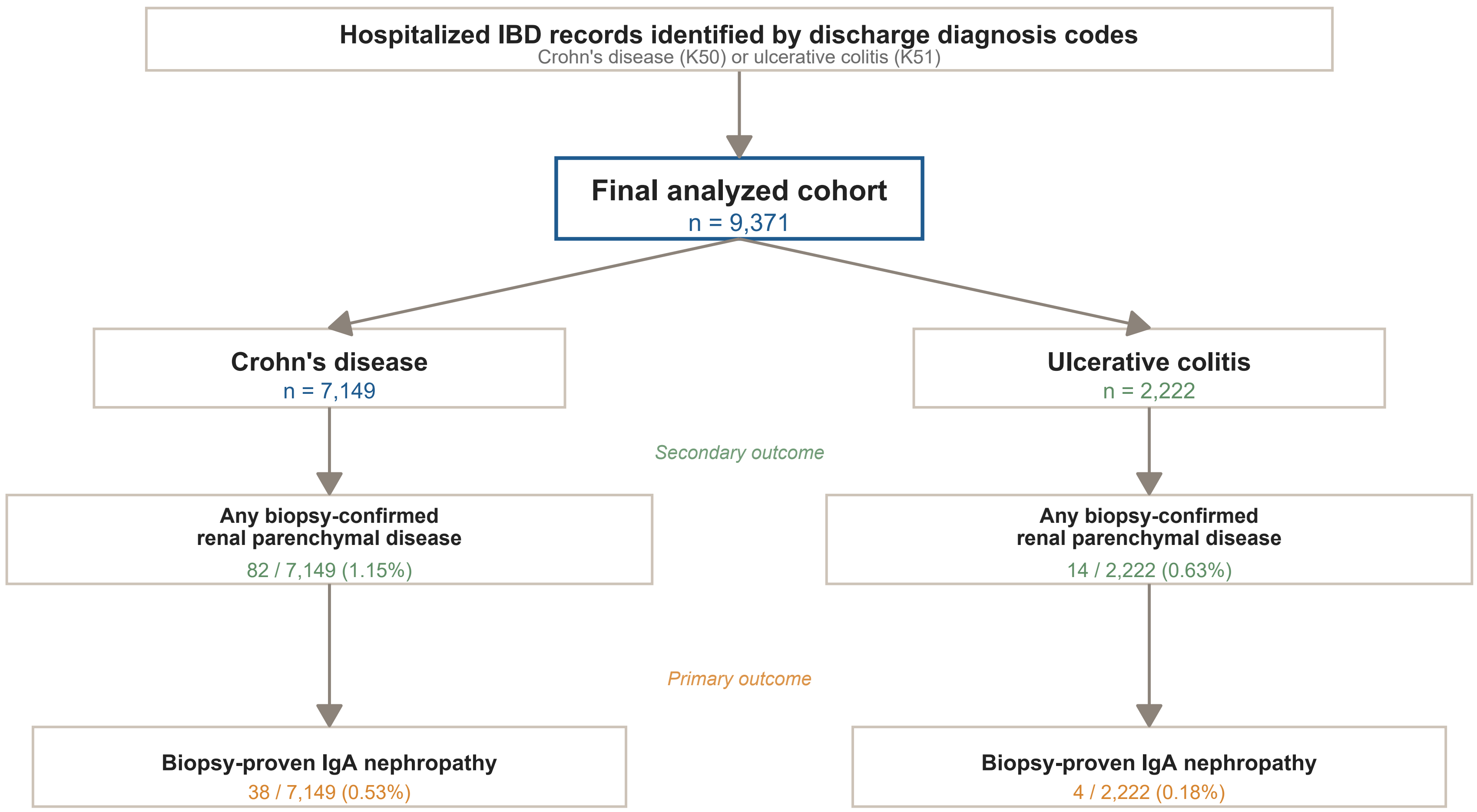

Renal parenchymal disease and IgA nephropathy were confirmed using kidney biopsy examination and diagnosis fields.

Total outcome counts: biopsy-confirmed renal parenchymal disease = 96; biopsy-proven IgA nephropathy = 42. Outcomes correspond to Table 1.

Supplementary Table S1. Summary of datasets and sample characteristics used in the study										
Note: Bidirectional two-sample MR used both European and East Asian disease GWAS datasets where available; all other genetic analyses used the European CD and European IgAN GWAS datasets unless otherwise specified.										
**Category**	**Dataset / trait / tissue**	**Source**	**Accession / PMID**	**Ancestry**	**Phenotype definition**	**Cases**	**Controls**	**Total N / samples**	**Genome build**	**Role in study**
**GWAS**	Inflammatory bowel disease	IIBDGC; accessed via IEU OpenGWAS	ieu-a-31; PMID: 26192919	European	Inflammatory bowel disease diagnosed based on accepted radiological, endoscopic, and histopathological evaluation, with fulfillment of clinical IBD criteria; cases included Crohn's disease	12,882	21,770	34,652	GRCh37/hg19	Bidirectional MR
**GWAS**	Crohn's disease	IIBDGC; accessed via IEU OpenGWAS	ieu-a-30; PMID: 26192919	European	Crohn's disease diagnosed based on accepted radiological, endoscopic, and histopathological evaluation, with fulfillment of clinical IBD criteria.	5,956	14,927	20,883	GRCh37/hg19	Primary CD analyses
**GWAS**	Ulcerative colitis	IIBDGC; accessed via IEU OpenGWAS	ieu-a-32; PMID: 26192919	European	Ulcerative colitis diagnosed based on accepted radiological, endoscopic, and histopathological evaluation, with fulfillment of clinical IBD criteria.	6,968	20,464	27,432	GRCh37/hg19	Bidirectional MR
**GWAS**	Inflammatory bowel disease	IIBDGC; accessed via NHGRI-EBI GWAS Catalog	GWAS Catalog accession: GCST90432182; PMID:37156999	East Asian	Inflammatory bowel disease diagnosed according to the European evidence-based consensus or ECCO–ESGAR diagnostic guidelines; cases included Crohn's disease and ulcerative colitis.	14,393	15,456	29,849	GRCh37/hg19	Bidirectional MR
**GWAS**	Crohn's disease	IIBDGC; accessed via NHGRI-EBI GWAS Catalog	GWAS Catalog accession: GCST90446793; PMID:37156999	East Asian	Crohn's disease diagnosed according to the European evidence-based consensus or ECCO–ESGAR diagnostic guidelines.	7,372	15,456	22,828	GRCh37/hg19	Bidirectional MR
**GWAS**	Ulcerative colitis	IIBDGC; accessed via NHGRI-EBI GWAS Catalog	GWAS Catalog accession: GCST90446795; PMID:37156999	East Asian	Ulcerative colitis diagnosed according to the European evidence-based consensus or ECCO–ESGAR diagnostic guidelines.	6,862	15,456	22,318	GRCh37/hg19	Bidirectional MR
**GWAS**	IgA nephropathy	Kiryluk Lab website (GWAS summary statistics)	European meta-analysis summary statistics; PMID: 37337107	European	Kidney-biopsy-diagnosed IgA nephropathy.	5,556	21,178	26,734	GRCh37/hg19	Primary IgAN analyses
**GWAS**	IgA nephropathy	Kiryluk Lab website (GWAS summary statistics)	East Asian meta-analysis summary statistics; PMID: 37337107	East Asian	Kidney-biopsy-diagnosed IgA nephropathy.	4,590	7,573	12,163	GRCh37/hg19	Bidirectional MR
**eQTL**	eQTLGen whole blood	eQTLGen	PMID:34475573	European	-	-	-	31,684	GRCh37/hg19	SMR; colocalization
**eQTL**	GTEx v8 Whole Blood	GTEx v8	PMID:32913098	Predominantly European	-	-	-	670	GRCh38	SMR; selected colocalization
**eQTL**	GTEx v8 Spleen	GTEx v8	PMID:32913098	Predominantly European	-	-	-	227	GRCh38	SMR; selected colocalization
**eQTL**	GTEx v8 Small Intestine - Terminal Ileum	GTEx v8	PMID:32913098	Predominantly European	-	-	-	174	GRCh38	SMR; selected colocalization
**eQTL**	GTEx v8 Colon - Sigmoid	GTEx v8	PMID:32913098	Predominantly European	-	-	-	318	GRCh38	SMR; selected colocalization
**eQTL**	GTEx v8 Colon - Transverse	GTEx v8	PMID:32913098	Predominantly European	-	-	-	368	GRCh38	SMR; selected colocalization
**eQTL**	GTEx v8 Lung	GTEx v8	PMID:32913098	Predominantly European	-	-	-	515	GRCh38	SMR; selected colocalization
**eQTL**	GTEx v8 Liver	GTEx v8	PMID:32913098	Predominantly European	-	-	-	208	GRCh38	SMR; selected colocalization
**scRNA-seq**	GSE134809 PBMC scRNA-seq subset from patients with ileal Crohn's disease	GEO; Martin et al., Cell, 2019	GSE134809; GSM4761136–GSM4761144; PMID: 31474370	Not explicitly reported	-	-	-	9	GRCh38	Cell-type localization

Total N/samples denotes the total sample size for GWAS and eQTL datasets and the number of donors for the scRNA-seq dataset. Only nine PBMC donors from GSE134809 (GSM4761136–GSM4761144) were included in the present analysis. Phenotype definitions were obtained from the corresponding source studies

Supplementary Table S2. Bidirectional two-sample MR, instrument characteristics, sensitivity statistics, and GSMR results

BH-FDR correction was applied separately to the six forward and six reverse IVW comparisons. Final estimates after MR-PRESSO outlier handling are reported where applicable.

A. Primary bidirectional two-sample MR results

Ancestry	Exposure	Outcome	Method	N SNPs	Beta	SE	P value	OR	OR 95% CI lower	OR 95% CI upper	BH-FDR
European	IBD	IgAN	IVW (random effects)	59	0.099	0.037	6.79E-03	1.105	1.028	1.187	1.36E-02
European	IBD	IgAN	MR-Egger	59	0.052	0.098	6.00E-01	1.053	0.869	1.276	
European	IBD	IgAN	Weighted median	59	0.022	0.044	6.12E-01	1.023	0.938	1.115	
European	IBD	IgAN	Weighted mode	59	0.005	0.059	9.30E-01	1.005	0.896	1.128	
European	IBD	IgAN	Simple mode	59	0.157	0.110	1.57E-01	1.170	0.944	1.451	
European	CD	IgAN	IVW (random effects)	49	0.066	0.029	2.48E-02	1.068	1.008	1.131	3.72E-02
European	CD	IgAN	MR-Egger	49	-0.018	0.064	7.77E-01	0.982	0.865	1.114	
European	CD	IgAN	Weighted median	49	0.018	0.037	6.24E-01	1.018	0.948	1.094	
European	CD	IgAN	Weighted mode	49	0.005	0.041	9.00E-01	1.005	0.927	1.090	
European	CD	IgAN	Simple mode	49	0.089	0.086	3.07E-01	1.093	0.923	1.293	
European	UC	IgAN	IVW (random effects)	34	0.068	0.042	1.04E-01	1.070	0.986	1.161	1.04E-01
European	UC	IgAN	MR-Egger	34	0.175	0.124	1.67E-01	1.191	0.935	1.519	
European	UC	IgAN	Weighted median	34	0.066	0.048	1.66E-01	1.069	0.973	1.174	
European	UC	IgAN	Weighted mode	34	0.039	0.069	5.71E-01	1.040	0.909	1.190	
European	UC	IgAN	Simple mode	34	0.095	0.107	3.81E-01	1.099	0.892	1.355	
European	IgAN	IBD	IVW (random effects)	3	0.037	0.070	5.99E-01	1.038	0.904	1.191	6.69E-01
European	IgAN	IBD	MR-Egger	3	-0.098	0.284	7.89E-01	0.907	0.520	1.582	
European	IgAN	IBD	Weighted median	3	0.008	0.040	8.41E-01	1.008	0.932	1.090	
European	IgAN	IBD	Weighted mode	3	0.004	0.037	9.18E-01	1.004	0.934	1.080	
European	IgAN	IBD	Simple mode	3	-0.018	0.052	7.59E-01	0.982	0.887	1.087	
European	IgAN	CD	IVW	3	-0.028	0.065	6.69E-01	0.973	0.856	1.105	6.69E-01
European	IgAN	CD	MR-Egger	3	-0.304	0.155	3.00E-01	0.738	0.545	0.999	
European	IgAN	CD	Weighted median	3	-0.025	0.053	6.40E-01	0.976	0.880	1.082	
European	IgAN	CD	Weighted mode	3	-0.082	0.057	2.88E-01	0.922	0.824	1.030	
European	IgAN	CD	Simple mode	3	0.128	0.127	4.19E-01	1.137	0.886	1.458	
European	IgAN	UC	IVW (random effects)	3	0.068	0.098	4.91E-01	1.070	0.882	1.298	6.69E-01
European	IgAN	UC	MR-Egger	3	-0.067	0.422	9.00E-01	0.935	0.409	2.139	
European	IgAN	UC	Weighted median	3	0.034	0.052	5.06E-01	1.035	0.935	1.146	
European	IgAN	UC	Weighted mode	3	0.026	0.054	6.77E-01	1.027	0.923	1.142	
European	IgAN	UC	Simple mode	3	-0.022	0.069	7.75E-01	0.978	0.854	1.119	
East Asian	IBD	IgAN	IVW	47	0.102	0.029	5.38E-04	1.107	1.045	1.173	3.23E-03
East Asian	IBD	IgAN	MR-Egger	47	0.158	0.071	3.13E-02	1.172	1.019	1.347	
East Asian	IBD	IgAN	Weighted median	47	0.111	0.039	4.37E-03	1.117	1.035	1.205	
East Asian	IBD	IgAN	Weighted mode	47	0.120	0.043	7.44E-03	1.127	1.037	1.226	
East Asian	IBD	IgAN	Simple mode	47	0.081	0.078	3.01E-01	1.084	0.932	1.263	
East Asian	CD	IgAN	IVW (random effects)	55	0.068	0.023	2.73E-03	1.071	1.024	1.120	8.19E-03
East Asian	CD	IgAN	MR-Egger	55	0.097	0.052	6.81E-02	1.101	0.995	1.219	
East Asian	CD	IgAN	Weighted median	55	0.096	0.031	1.87E-03	1.100	1.036	1.169	
East Asian	CD	IgAN	Weighted mode	55	0.089	0.032	7.72E-03	1.093	1.026	1.163	
East Asian	CD	IgAN	Simple mode	55	0.068	0.048	1.69E-01	1.070	0.973	1.176	
East Asian	UC	IgAN	IVW	19	0.067	0.038	7.62E-02	1.069	0.993	1.152	9.15E-02
East Asian	UC	IgAN	MR-Egger	19	0.128	0.175	4.75E-01	1.137	0.806	1.603	
East Asian	UC	IgAN	Weighted median	19	0.031	0.055	5.75E-01	1.032	0.926	1.150	
East Asian	UC	IgAN	Weighted mode	19	0.032	0.083	7.04E-01	1.033	0.877	1.216	
East Asian	UC	IgAN	Simple mode	19	0.062	0.099	5.42E-01	1.064	0.875	1.293	
East Asian	IgAN	IBD	IVW	9	-0.058	0.049	2.41E-01	0.944	0.857	1.040	6.69E-01
East Asian	IgAN	IBD	MR-Egger	9	-0.311	0.305	3.42E-01	0.733	0.403	1.332	
East Asian	IgAN	IBD	Weighted median	9	-0.069	0.054	2.05E-01	0.933	0.839	1.038	

East Asian	IgAN	IBD	Weighted mode	9	-0.085	0.074	2.80E-01	0.918	0.795	1.061	
East Asian	IgAN	IBD	Simple mode	9	-0.068	0.087	4.57E-01	0.934	0.788	1.108	
East Asian	IgAN	CD	IVW	11	-0.040	0.040	3.25E-01	0.961	0.888	1.040	6.69E-01
East Asian	IgAN	CD	MR-Egger	11	0.218	0.147	1.74E-01	1.243	0.931	1.660	
East Asian	IgAN	CD	Weighted median	11	-0.004	0.054	9.41E-01	0.996	0.895	1.108	
East Asian	IgAN	CD	Weighted mode	11	0.054	0.068	4.46E-01	1.055	0.924	1.205	
East Asian	IgAN	CD	Simple mode	11	-0.018	0.097	8.58E-01	0.982	0.813	1.188	
East Asian	IgAN	UC	IVW (random effects)	9	-0.074	0.083	3.70E-01	0.928	0.789	1.092	6.69E-01
East Asian	IgAN	UC	MR-Egger	9	-0.187	0.546	7.42E-01	0.830	0.285	2.418	
East Asian	IgAN	UC	Weighted median	9	-0.082	0.077	2.87E-01	0.921	0.792	1.071	
East Asian	IgAN	UC	Weighted mode	9	-0.235	0.157	1.73E-01	0.790	0.581	1.076	
East Asian	IgAN	UC	Simple mode	9	0.161	0.198	4.41E-01	1.174	0.796	1.731	

B. Heterogeneity and MR-Egger intercept statistics

Ancestry	Exposure	Outcome	N SNPs	Cochran's Q	Q df	Q P value	MR-Egger intercept	Intercept SE	Intercept P value	MR-PRESSO global P value
European	IBD	IgAN	59	114.517	57	9.71E-06	0.008	0.016	0.60	<1.00E-04
European	CD	IgAN	49	86.081	47	4.39E-04	0.020	0.014	0.15	3.00E-04
European	UC	IgAN	34	64.453	32	5.82E-04	-0.021	0.023	0.36	1.00E-03
European	IgAN	IBD	3	6.161	1	1.31E-02	0.046	0.091	0.71	Not applicable
European	IgAN	CD	3	0.036	1	8.49E-01	0.093	0.050	0.31	Not applicable
European	IgAN	UC	3	8.534	1	3.49E-03	0.046	0.136	0.79	Not applicable
East Asian	IBD	IgAN	47	56.684	45	1.14E-01	-0.011	0.013	0.39	1.36E-01
East Asian	CD	IgAN	55	78.930	53	1.20E-02	-0.008	0.013	0.55	1.67E-02
East Asian	UC	IgAN	19	17.596	17	4.15E-01	-0.013	0.035	0.73	4.63E-01
East Asian	IgAN	IBD	9	11.822	7	1.07E-01	0.051	0.060	0.43	1.40E-01
East Asian	IgAN	CD	11	8.267	9	5.08E-01	-0.059	0.033	0.10	2.68E-01
East Asian	IgAN	UC	9	23.337	7	1.49E-03	0.023	0.112	0.84	3.90E-03

MR-PRESSO was not performed for analyses with fewer than four instrumental variants.

C. GSMR results

Ancestry	Exposure	Outcome	Method	N SNPs	Beta	SE	P value
European	CD	IgAN	GSMR	84	0.235	0.057	4.02E-05
European	IgAN	CD	GSMR	11	-0.369	0.154	1.65E-02

The reverse GSMR analysis included 11 instruments versus 84 in the forward analysis; the reverse estimate was therefore less precise.

D. Instrument selection, strength, and directionality

Ancestry	Exposure	Outcome	SNPs before clumping	SNPs after LD clumping	SNPs after harmonization	MR-PRESSO outliers removed	Final instruments	Minimum F	Mean F	Approximate cumulative R^2 (%)	Steiger-consistent SNPs
European	IBD	IgAN	10299	64	60	1	59	29.81	62.02	10.53	59/59
European	CD	IgAN	5237	53	49	0	49	29.97	58.70	13.71	49/49
European	UC	IgAN	6616	38	35	1	34	29.71	60.50	7.47	34/34
European	IgAN	IBD	2647	6	3	0	3	39.76	50.30	0.56	3/3
European	IgAN	CD	2647	6	3	0	3	39.76	50.30	0.56	3/3
European	IgAN	UC	2647	6	3	0	3	39.76	50.30	0.56	3/3
East Asian	IBD	IgAN	11154	58	50	3	47	30.14	67.02	10.50	47/47
East Asian	CD	IgAN	12360	62	57	2	55	29.84	68.81	16.44	55/55
East Asian	UC	IgAN	13994	31	23	4	19	31.38	52.19	4.43	19/19
East Asian	IgAN	IBD	3331	16	12	3	9	30.15	43.33	3.19	9/9
East Asian	IgAN	CD	3331	16	11	0	11	30.15	58.62	5.27	11/11
East Asian	IgAN	UC	3331	16	12	3	9	30.15	42.67	3.15	9/9

LD clumping used $r^2 < 0.001$ within a 10,000-kb window. Ambiguous palindromic variants were excluded, with no proxy variants used. Final instruments were retained after harmonization and MR-PRESSO outlier removal; all had $F > 10$.

Supplementary Table S3. Local genetic architecture and cross-trait locus evidence from LAVA, PLACO, and MTAG

Section A lists Bonferroni-significant LAVA local genetic-correlation blocks. Section B lists independent potential additional shared loci retained after PLACO significance filtering and LD-based exclusion, with original GWAS and MTAG P values reported for each locus. Section C reports MTAG diagnostic statistics and covariance estimates. Section D summarizes PLACO analysis parameters and locus-retention criteria.

A. LAVA-significant local genetic-correlation blocks

Region	Chr	Start (bp)	Stop (bp)	n SNPs	Local rg (rho)	95% CI	P value	Direction	CD local h2	IgAN local h2
chr5 (95.1–96.5 Mb)	5	95117260	96467377	2952	0.635	0.384 to 0.918	5.01E-06	Positive	0.008	0.005
chr9 (117.1–118.0 Mb)	9	117054910	117989017	2506	0.682	0.445 to 0.981	6.83E-07	Positive	0.012	0.004
chr10 (80.0–81.2 Mb)	10	79952997	81190573	3050	0.690	0.422 to 1.000	4.26E-06	Positive	0.007	0.007
chr11 (75.4–76.5 Mb)	11	75445254	76518906	2107	0.651	0.383 to 0.965	1.17E-05	Positive	0.008	0.005
chr18 (71.5–72.8 Mb)	18	71491861	72785206	3014	-0.976	-1.000 to -0.543	4.02E-05	Negative	0.006	0.002

B. Independent potential additional shared loci retained after PLACO filtering and LD-based exclusion

Lead SNP	Chr	Position (bp)	PLACO P	CD GWAS P	IgAN GWAS P	CD MTAG P	IgAN MTAG P	Within significant LAVA block
rs12748081	1	172867006	2.86E-11	1.34E-12	3.38E-04	7.49E-14	5.91E-12	No
rs10041894	5	40493234	2.65E-09	6.88E-14	5.94E-03	1.06E-14	8.99E-11	No
rs2617439	5	96275154	5.41E-14	7.48E-12	2.71E-06	1.23E-13	3.33E-14	Yes: chr5 (95.1–96.5 Mb)
rs4921224	5	158804182	4.76E-10	6.89E-15	3.96E-03	7.36E-16	1.29E-11	No
rs10046001	5	158814577	5.29E-11	9.79E-17	2.67E-03	1.05E-17	8.38E-13	No
rs2515919	6	31564167	1.83E-13	4.38E-09	1.67E-07	5.77E-11	1.13E-13	No
rs6478108	9	117558703	2.52E-11	1.69E-12	3.03E-04	8.78E-14	5.54E-12	Yes: chr9 (117.1–118.0 Mb)
rs36118932	9	117616367	3.89E-11	1.36E-07	2.51E-06	4.05E-09	2.38E-11	Yes: chr9 (117.1–118.0 Mb)
rs1250573	10	81042475	8.30E-10	9.01E-11	8.14E-04	7.91E-12	2.24E-10	Yes: chr10 (80.0–81.2 Mb)
rs6584283	10	101290301	1.37E-09	8.68E-19	1.49E-02	1.65E-19	1.72E-12	No
rs34452117	18	12784686	1.51E-10	2.84E-11	4.14E-04	1.76E-12	4.28E-11	No

Within significant LAVA block indicates positional overlap with the Bonferroni-significant LAVA intervals listed in Section A. PLACO P values are nominal; all listed variants had Bonferroni-adjusted P values < 0.05.

C. MTAG diagnostic statistics and covariance estimates

Trait	SNPs used	Original GWAS N	GWAS mean χ^2	MTAG mean χ^2	GWAS-equivalent N	Maximum FDR
CD	5,700,438	20,883	1.175	1.179	21,438	0.019
IgAN	5,700,438	26,734	1.034	1.057	44,781	0.232

MTAG genetic and error covariance matrices

Estimated Ω	CD	IgAN
CD	8.551E-06	1.304E-06
IgAN	1.304E-06	1.296E-06

Estimated Σ	CD	IgAN
CD	1.022	8.00E-03
IgAN	0.008	1.018

Ω denotes the estimated genetic covariance matrix and Σ denotes the estimated error covariance matrix. The corresponding CD–IgAN correlations were 0.392 for Ω and 0.007 for Σ.

D. PLACO analysis parameters and locus-retention criteria

Item	Parameter / criterion
PLACO software	The original PLACO R script (v0.2.0) was used.
Significance threshold	PLACO significance was defined as a Bonferroni-adjusted P value < 0.05 across 7,352,033 tested variants, corresponding to a nominal PLACO P value $< 6.80 \times 10^{-9}$.
Handling of Z statistics	Variants with squared Z scores greater than 80 for either trait were excluded, and the remaining trait-specific Z scores were decorrelated before PLACO testing.
Potential sample overlap	Potential sample overlap was evaluated using the LDSC cross-trait intercept, and cross-trait correlation in the Z statistics was addressed through decorrelation before PLACO testing.
Initial LD clumping	PLACO-significant variants were clumped using the European 1000 Genomes reference panel with a 500-kb window and $r^2 < 0.3$.
Definition of a known shared locus	A variant was defined as a known shared locus when it reached genome-wide significance ($P < 5 \times 10^{-8}$) in both original CD and IgAN GWAS.
Known-locus LD exclusion	Variants within the LD range of known shared loci were identified using an expanded 1,000-kb window and $r^2 < 0.3$ and were excluded.
Final locus-retention criterion	The remaining independent PLACO-significant variants that did not reach genome-wide significance in at least one original GWAS were retained as potential additional shared loci.

Supplementary Table S4. Regional colocalization, SMR effect estimates, and gene-level results at the ERAP2 and CARD9 loci

A. Regional GWAS–GWAS colocalization

Region / locus	Evidence	Disease comparison	GWAS–GWAS PPH4 (%)
chr9:117.1–118.0 Mb	LAVA-significant block	CD vs IgAN	42.0
chr10:80.0–81.2 Mb	LAVA-significant block	CD vs IgAN	44.8
chr5:95.1–96.5 Mb	LAVA-significant block	CD vs IgAN	88.4
chr11:75.4–76.5 Mb	LAVA-significant block	CD vs IgAN	3.0
chr18:71.5–72.8 Mb	LAVA-significant block	CD vs IgAN	0.6
chr9 CARD9 region	Gene-centered regional colocalization	CD vs IgAN	98.4

B. SMR effect estimates for ERAP2 and CARD9

Region / locus	Gene	Disease	eQTL source	Tissue	Top cis-eQTL SNP	EA	OA	eQTL β	GWAS β	SMR β	SMR P	HEIDI P	HEIDI nSNP
chr5 ERAP2 locus	ERAP2	CD	eQTLGen	Whole blood	rs2910686	C	T	1.011900	0.149696	0.147936	6.501E-11	1.024E-01	8
chr5 ERAP2 locus	ERAP2	CD	GTEx v8	Spleen	rs2910686	C	T	1.171860	0.149696	0.127742	5.363E-10	2.270E-01	5
chr5 ERAP2 locus	ERAP2	CD	GTEx v8	Small intestine – Terminal ileum	rs2910686	C	T	1.019990	0.149696	0.146762	1.445E-09	4.602E-01	5
chr5 ERAP2 locus	ERAP2	CD	GTEx v8	Colon – Sigmoid	rs2927608	A	G	1.037960	0.150100	0.144611	4.080E-10	2.664E-01	5
chr5 ERAP2 locus	ERAP2	CD	GTEx v8	Colon – Transverse	rs2927608	A	G	1.052830	0.150100	0.142568	2.802E-10	5.235E-01	5
chr5 ERAP2 locus	ERAP2	CD	GTEx v8	Lung	rs2927608	A	G	1.219240	0.150100	0.123109	8.806E-11	4.646E-01	5
chr9 CARD9 locus	CARD9	CD	eQTLGen	Whole blood	rs11794847	A	G	0.590152	0.211103	0.357710	4.775E-19	4.653E-02	20
chr9 CARD9 locus	CARD9	IgAN	eQTLGen	Whole blood	rs11794847	A	G	0.590152	0.153600	0.260272	5.766E-10		
chr9 CARD9 locus	CARD9	CD	GTEx v8	Lung	rs4075760	G	A	0.169130	0.211499	1.250510	1.952E-08	5.226E-01	20
chr9 CARD9 locus	CARD9	IgAN	GTEx v8	Lung	rs4075760	G	A	0.169130	0.152900	0.904038	2.726E-06		

C. GWAS–eOTL colocalization

Region / locus	Gene	Disease	eQTL source	Tissue	Representative SNP	EA	OA	eQTL β	GWAS β	n SNPs	Coloc PPH4 (%)
chr5 ERAP2 locus	ERAP2	CD	eQTLGen	Whole blood	rs6873866	C	T	-1.03782	-0.16810	7,479	72.4
chr5 ERAP2 locus	ERAP2	IgAN	eQTLGen	Whole blood	rs248215	A	G	-1.09603	-0.14730	5,931	72.7
chr5 ERAP2 locus	ERAP2	CD	GTEx v8	Colon – Sigmoid	rs2927608	A	G	1.03796	0.15010	6,302	75.7
chr5 ERAP2 locus	ERAP2	IgAN	GTEx v8	Colon – Sigmoid	rs2927608	A	G	1.03796	0.13990	5,746	90.4
chr5 ERAP2 locus	ERAP2	CD	GTEx v8	Colon – Transverse	rs2927608	A	G	1.05283	0.15010	6,302	76.4
chr5 ERAP2 locus	ERAP2	IgAN	GTEx v8	Colon – Transverse	rs2927608	A	G	1.05283	0.13990	5,746	87.3
chr5 ERAP2 locus	ERAP2	CD	GTEx v8	Small intestine – Terminal ileum	rs2910686	C	T	1.01999	0.14970	6,301	77.0
chr5 ERAP2 locus	ERAP2	IgAN	GTEx v8	Small intestine – Terminal ileum	rs2927608	A	G	1.01999	0.13990	5,746	85.0
chr9 CARD9 locus	CARD9	CD	eQTLGen	Whole blood	rs4077515	T	C	0.74685	0.21590	8,359	88.0
chr9 CARD9 locus	CARD9	IgAN	eQTLGen	Whole blood	rs4077515	T	C	0.74685	0.15510	5,424	88.9
chr9 CARD9 locus	CARD9	CD	GTEx v8	Spleen	rs4266763	G	A	0.16453	0.21180	6,603	59.3
chr9 CARD9 locus	CARD9	CD	GTEx v8	Small intestine – Terminal ileum	rs4077515	T	C	0.03343	0.21590	6,602	3.7

D. CD-side SMR results for genes at the 5q15 locus using eOTLGen whole-blood eOTL data

Gene	Ensembl ID	Top cis-eQTL SNP	EA/OA	eQTL β	CD GWAS β	SMR β	SMR P	HEIDI P	HEIDI nSNP
CAST	ENSG00000153113	rs55770741	C/T	-0.150530	0.148500	-0.986514	2.015E-09	2.547E-02	8
ERAP1	ENSG00000164307	rs18059	T/C	0.087036	-0.126198	-1.449950	8.525E-07	2.372E-02	8
ERAP2	ENSG00000164308	rs2910686	C/T	1.011900	0.149696	0.147936	6.501E-11	1.024E-01	8
LNPEP	ENSG00000113441	rs2617439	A/G	0.312487	0.158797	0.508172	1.766E-11	1.996E-02	8

EA, effect allele; OA, other allele. All β estimates are aligned to the EA. The top cis-eQTL SNP denotes the lead cis-eQTL instrument used in the SMR analysis. HEIDI P > 0.05 was considered evidence against substantial heterogeneity. No gene-level SMR result at the 5q15 locus met the prespecified criteria in the IgAN-side analysis.

Supplementary Table S5. Operational definitions of exposure, renal outcomes, and covariates

Retrospective clinical cohort, The Sixth Affiliated Hospital, Sun Yat-sen University (2012-10-01 to 2025-10-31)

Variable	Source field(s)	Definition
**Study cohort**	Discharge diagnosis records	Hospitalized inflammatory bowel disease records identified using discharge diagnosis codes for Crohn's disease (K50) or ulcerative colitis (K51).
**IBD subtype**	IBD subtype	Crohn's disease or ulcerative colitis.
**Biopsy-proven IgA nephropathy**	Kidney biopsy examination field; kidney biopsy diagnosis field	IgA nephropathy confirmed by kidney biopsy examination and diagnosis records.
**Any biopsy-confirmed renal parenchymal disease**	Kidney biopsy examination field; kidney biopsy diagnosis field	Any renal parenchymal disease confirmed by kidney biopsy, including biopsy-proven IgA nephropathy and other histopathologically confirmed renal parenchymal lesions. Nephrolithiasis, isolated renal dysfunction without pathological confirmation, and non-parenchymal or urological conditions were excluded.
**Age**	Age	Age at index IBD hospitalization.
**Sex**	Sex	Male or female.

Abbreviations: IBD, inflammatory bowel disease.
